# Ranking Regions, Edges and Classifying Tasks in Functional Brain Graphs by Sub-Graph Entropy

**DOI:** 10.1038/s41598-019-44103-8

**Published:** 2019-05-20

**Authors:** Bhaskar Sen, Shu-Hsien Chu, Keshab K. Parhi

**Affiliations:** Department of Electrical and Computer Engineering, University of Minnesota - Twin Cities, Minneapolis, USA

**Keywords:** Data mining, Machine learning, Statistical methods, Network models, Electrical and electronic engineering

## Abstract

This paper considers analysis of human brain networks or graphs constructed from time-series collected from functional magnetic resonance imaging (fMRI). In the network of time-series, the nodes describe the regions and the edge weights correspond to the absolute values of correlation coefficients of the time-series of the two nodes associated with the edges. The paper introduces a novel information-theoretic metric, referred as *sub-graph* entropy, to measure uncertainty associated with a *sub-graph*. Nodes and edges constitute two special cases of *sub-graph* structures. Node and edge entropies are used in this paper to rank regions and edges in a functional brain network. The paper analyzes task-fMRI data collected from 475 subjects in the Human Connectome Project (HCP) study for gambling and emotion tasks. The proposed approach is used to rank regions and edges associated with these tasks. The *differential* node (edge) entropy metric is defined as the difference of the node (edge) entropy corresponding to two different networks belonging to two different classes. Differential entropy of nodes and edges are used to rank top regions and edges associated with the two classes of data. Using top node and edge entropy features separately, two-class classifiers are designed using support vector machine (SVM) with radial basis function (RBF) kernel and leave-one-out method to classify time-series for emotion task *vs.* no-task, gambling task *vs.* no-task and emotion task *vs*. gambling task. Using node entropies, the SVM classifier achieves classification accuracies of 0.96, 0.97 and 0.98, respectively. Using edge entropies, the classifier achieves classification accuracies of 0.91, 0.96 and 0.94, respectively.

## Introduction

The *state* of the human brain network changes dynamically from task to task or from resting-state to a task, where each state represents a specific pattern in brain connectivity. Finding patterns in those connectivity *states* are of utmost *importance*^1,2^. Recently there has been a surge of interest in understanding brain connectivity patterns while a person performs a task through the use of network theory^3–5^. Although complex network measures have been applied previously to analyze brain networks, several areas within this particular sub-field remain unexplored. This paper introduces the notions of graph entropy and *sub-graph* entropy and applications of these metrics to functional brain network analysis and classification. We propose the use of *sub-graph* entropy as an information-theoretic measure to compute complexity of brain networks. Special cases of *sub-graph* entropy include node entropy and edge entropy. It may be noted that, to the authors’ best knowledge, this is the first attempt to make use of *sub-graph* entropy to analyze brain networks. We also propose ranking of regions and edges of functional brain networks using these metrics. Node entropy and edge entropy are used as features for classifying *functional connectivity* patterns from task-fMRI (t-fMRI) corresponding to a number of unique *states*. The t-fMRI data is taken from emotion and gambling tasks from Human Connectome Project (HCP) dataset^6^. Although, emotion and gambling tasks are used in this paper for illustration, the proposed information-theoretic metric is generalizable to other tasks and potentially to two different groups, *e*.*g*., patients *vs*. controls, male *vs*. female etc.

There are multiple ways to define brain connectivity. *Structural connectivity* refers to a range of physical links that connect neuronal units. *Functional connectivity* captures patterns of deviations from statistical independence between distributed and possibly distant neuronal units^7,8^. Joint connectivity captures links that connect neuronal units both physically and functionally^9^. Among these, *functional connectivity* is highly time dependent, and it can be statistically nonstationary. It is modulated by external task demands and sensory stimulation, as well as the internal *state* of the organism. In this paper, we use *functional connectivity* extracted from t-fMRI as representative of brain *states*. In this representation, each region is a collection of neural elements, defined based on anatomy of brain tissues^10^. The brain activities of each region are represented by different time-series corresponding to different voxels and their average value can represent the behavior of the region over time. The functional brain network (graph) is represented by nodes and edges, where each node is associated with the mean time-series of a brain region and each edge weight corresponds to the *absolute value* of the correlation coefficient of the two time-series of the two vertices of the edge. This view is popular in fMRI literature and finds evidence through the works of^1,11^.

Task-fMRI studies of human brain have previously focused on finding a representative network connectivity corresponding to a *state*^2^. The application of network theory for analyzing the *states* has revealed that individual human brain exhibits centrality property^3^, i.e., some human brain regions have higher *importance* in the whole network than others in terms of connections to other nodes. The centrality properties of a network are utilized to infer information about the *state*. For example, if a node has high *centrality* value in a network, the corresponding *state* can be understood in terms of behavior of the node. Although, these network metrics are well suited to extract regions based on a particular definition of *importance*, how these measures can be applied to classify two *states* from brain connectivity networks remains unclear. We believe that information-theoretic approaches can be useful to address this challenge. We introduce information-theoretic entropy measures for analyzing and classifying brain networks in this paper.

During the emotion and gambling t-fMRI experiment, a subject performs the task in blocks. The time periods when a task is performed are interleaved by rest periods. Hence from each subject, two functional networks each corresponding to a particular *state* (*e*.*g*., task *vs*. no-task for emotion and gambling) of brain are extracted. For simplicity, we assume that the brain *state* remains similar for the whole duration of task or no-task. Hence each functional network can be seen as representative of that particular *state*.

### Task State Network

The network connectivity for a task is constructed, by taking the absolute Pearson correlation coefficient between anatomical regions from fMRI time points, when a subject is performing a task (*e*.*g*., emotion, gambling) during a t-fMRI experiment^12–15^. Note that each subject has one network corresponding to a task *state*. In this paper, t-fMRI time-series have been extracted from 475 subjects for emotion and gambling task from the Human Connectome Project^16^.

### No-task State Network

The network connectivity for a no-task is constructed from fMRI time points when a subject is *not* performing a task during a t-fMRI experiment. These time points contain baseline hemodynamic signals during transition periods^17^. Note that each subject has one network corresponding to no-task *state*.

## Hypothesis and Contributions

The main objective of the study is to understand whether brain *states* can be predicted using network measures from t-fMRI. First, we hypothesize that there are *important* nodes and edges in *functional connectivity*, that have significantly different network centrality measures for two different brain *states*. Our second hypothesis is that incorporating the *important* nodes, edges, and the corresponding centrality metrics to a classification model will lead to better prediction accuracy. Additionally, we hypothesize that the network metric, which is most predictive of two *states*, will also be group differentiating and biologically meaningful. How the hypotheses are analyzed is described next.

### Analysis of Hypothesis 1: Importance of Nodes and Edges

Using the information-theoretic network measures, we provide a novel way to identify *important* regions and edges from a network. Here, *important* regions (respectively, edges) are defined as the ones containing maximum entropy in *sub-graph*s. Also, how the regional *importance* changes between task *vs*. no-task or two different task conditions (emotion and gambling) is demonstrated. The *important* regions and edges extracted by this process are shown to be meaningful for classifying brain states.

### Analysis of Hypothesis 2: Classifying Two Brain States

**Figure 1:**
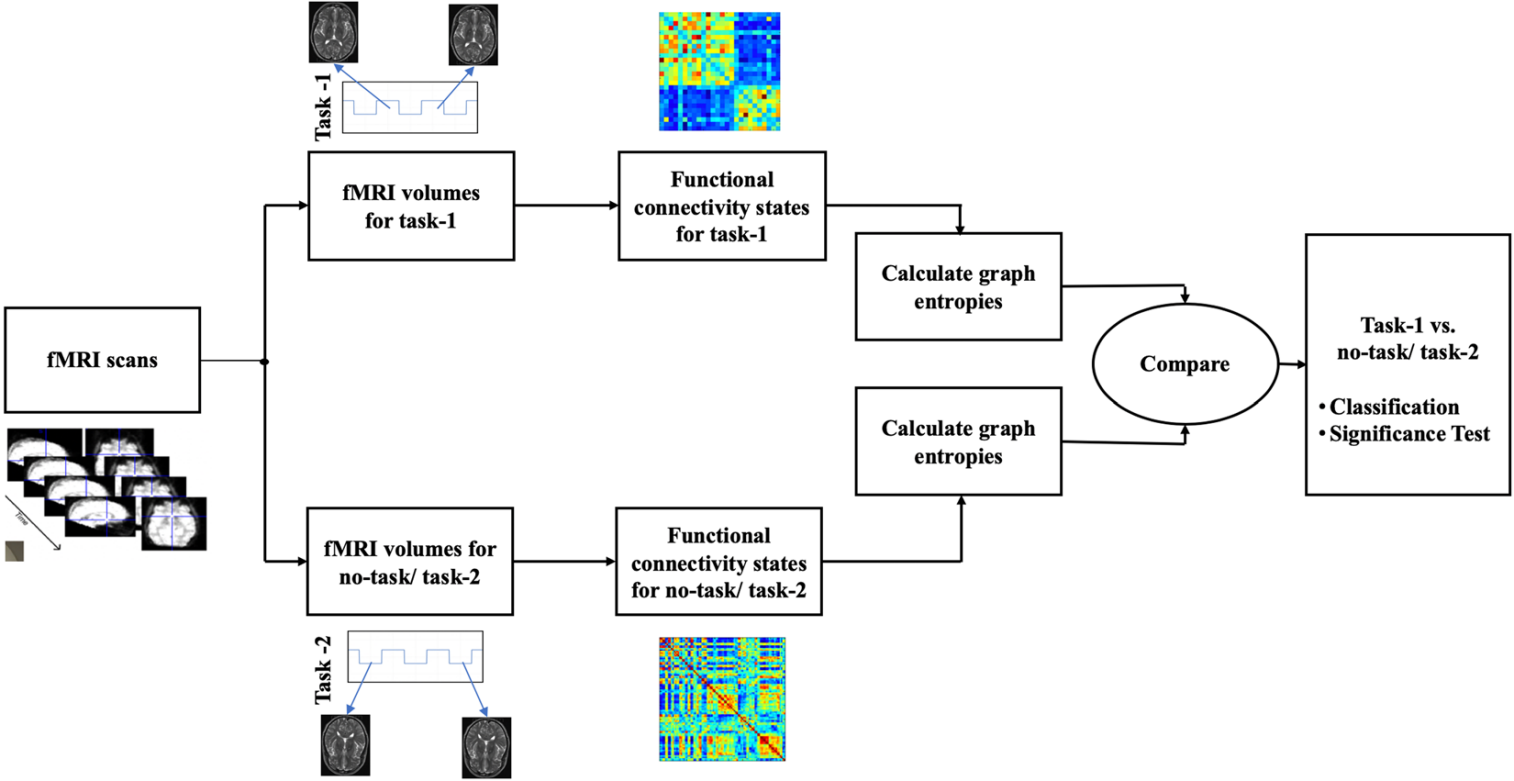
Pipeline for comparing group level entropy differences. After parcellating fMRI scans into regions, graph entropies are calculated for each subject’s functional network. These entropies are then used to compare two different *states*.

We use *sub-graph* entropy to characterize each of the *states* in a quantitative way. After the functional network is extracted, the entropies between two different groups of networks are compared. Binary classifiers are designed using the proposed and state-of-the-art network metrics to classify two *states*. The analysis pipeline for this is showed in Fig. 1. Classification accuracy and group statistical test (t-test and effect size) are computed for the entropy values to demonstrate that they are statistically different for different task conditions. The classification performance is also compared with previously known state-of-the-art network centrality metrics.

### Contributions

Contributions of this paper are three-fold. First, we propose the use of node and edge entropies as centrality metrics to compute the complexity of brain networks. Second, we propose ranking of nodes and edges of brain networks to extract *important* regions and edges between two *states*. We show that *sub-graph* entropy extracts some *important* regions, that the other network metrics can not identify. Third, using emotion and gambling t-fMRI data from the HCP dataset, we show that there is statistical difference of entropy measure between task *state vs*. no-task *state* and between two tasks. Node entropy and edge entropy are used as features to classify task *vs*. no-task or two different tasks with high accuracy. The proposed method outperforms other centrality measures for classifying two *states*. This validates the efficacy of *sub-graph* entropy as neural correlates of *states*.

## Previous Work

Several metrics have been proposed in the neuroimaging literature^11,18,19^ to compare different brain *states*. In this paper, we represent each brain *state* as a network corresponding to different induced conditions as described before^12–15,20,21^. This section describes previous works on analysing brain network (corresponding to *states*) based on regional and edge *importance*.

### Node Importance

In a complex network, different nodes may have different usages. Some may be used more than others, whereas some nodes might be controlling the dynamics of the whole network. These measures describe the centrality properties of the graph^22^. Statistical significance tests are commonly used to infer about the most important regions and links associated with an external stimulation. Here we describe a statistical way to infer about important regions during task *states* using generalized linear models (GLM). Among the network-theoretic measures commonly used to infer about important nodes, we illustrate four centrality measures, namely, *degree* centrality, *eigenvector* centrality, *betweenness* centrality and *leverage* centrality. Generalized linear models (GLM)^23^ use multiple regression with false discovery rate controls to infer the most important regions during a task. *Degree* centrality^24^ defines the central nodes to be the ones having the highest number of connections with other nodes. This centrality metric computes the *importance* of a node in the network by just the number of other nodes with which it directly interacts. *Eigenvector* centrality^25^ takes into account the centrality of immediate neighbors when computing the centrality of a particular node. In particular, *eigenvector* centrality is a positive multiple of the sum of nearest node centralities. They are computationally very intensive compared to the other centrality metrics. *Betweenness* centrality^26^ of a node represents the *importance* from the perspective of shortest paths in a graph. Particularly, this metric is calculated as the fraction of the shortest paths between all pairs of nodes (except the node in consideration) of a graph that contain the given node^27^. Joyece *et al*.^22^ introduced a new measure of centrality called *leverage* centrality that finds out the influence of a node in a graph on other neighboring nodes based on their degree distribution. However, we note that the centrality measures may not only depend on degrees but also on the weight of the link between them. For example, if the weight of an edge is higher, it is more likely to be used. The information of the edge weights can be used to develop a new *importance* measure. In addition, all these centrality measures are only applicable when the topological structure of network is clearly known for every individual sample. In stochastic networks where the group behavior of a number of networks is of utmost *importance*, the extension of these measures is not straight-forward for differentiation between two groups. More details about these network measures can be found in^3^.

### Edge Importance

There have been a few previous works for understanding the *importance* of edges in brain *states*. Among them, Network Based Statistics (NBS)^28^ is a popular method for testing hypotheses about the edges in a network using t-test. It is used to identify connections and networks comprising the connectome associated with an experiment for a between-group difference.

### Node and Edge Importance to Predict Brain States

This paper introduces an information-theoretic approach to bridge the gap of understanding node and edge *importance* from brain networks (corresponding to *states*) to classify *states*. Here we note that information-theoretic centrality metrics have been proposed before, although in a different setting. Information-theoretic approaches have been used in communication engineering since the seminal paper^29^ of Shannon in 1949. The information-theoretic concepts have been applied to understand different types of complex systems, *e*.*g*., in chemical graph theory^30,31^. From a structural complexity viewpoint, graph entropy was first introduced by Trucco^32^ and later formalized by Mowshowitz^33^. Structural graph entropy based on information-theoretic functional was introduced in^34^. The view of defining entropy based on intra-network communication between nodes was introduced in^35^. Mackenzie^35^ showed that information-theoretic *importance* can be used as centrality in a communication network. Shetty *et al*.^36^ defined an information-theoretic centrality measure^37^ to find out leaders and followers from a communication pattern between employees of an organization. This formulation considered the communication pattern over a number of days between agents to infer their *importance*. An integrated approach for understanding node, edge *importance* and using them for prediction have never been accomplished before.

## Results

This section proposes the information-theoretic metrics for analysing networks in order to extract important nodes and edges. It also demonstrates the classification results of applying node entropy and edge entropy to two different conditions on human brain networks. First, graph entropy, *sub-graph* entropy, node entropy and edge entropy are illustrated using a simple example. Second, important regions and edges based on change in group (node and edge) entropy are ranked. Third, node and edge entropy values are used to design classifiers for classifying two connectivity *states* for emotion and gambling tasks. The classification performance is compared with the state-of-the-art network metrics for classification of *states*. The performance is also compared with a recently developed tensor based model for task prediction. Fourth, we compare *graph entropy* based centrality measure with commonly used centrality measures like *degree*, *betweenness*, *eigenvector and leverage*. A comparison of graph entropy based centrality with structural centrality is also shown in Subsection S.7 and Fig. S13 in Supplementary Information. In addition, regions found through graph entropy are compared with the ones extracted by GLM and NBS. Lastly, the group-level differences of whole brain network between task *vs*. no-task (or task 1 *vs*. task 2) are investigated.

The brain region parcellation is based on^38^. In this paper, for all subsequent brain networks, we use the regions of interest (85 in total) as defined in^38^ viewed with BrainNet Viewer^39^.

### Illustration on Graph Entropy

**Figure 2:**
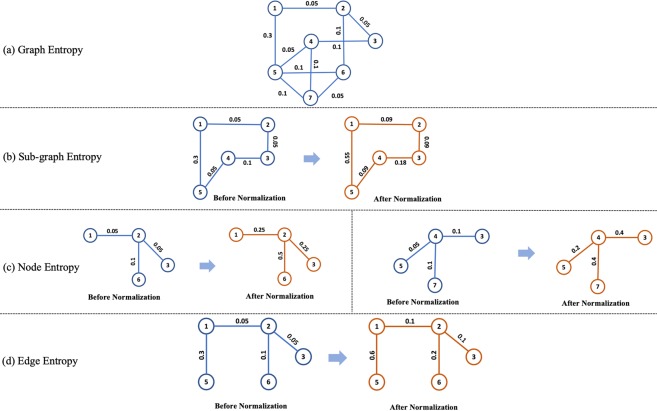
(**a**) An example of calculating graph entropy. This graph consists of 7-nodes. The weighted edges between them are normalized, i.e., they sum up to 1. (**b**) An example of *sub-graph* from the example in (**a**). To calculate the *sub-graph* entropy, we normalize the edge connection. *Left: sub-graph* before normalization. *Right: sub-graph* after normalization. This *sub-graph* consists of 5-nodes. As the weighted edge between them are normalized, they sum up to 1. (**c**) *Sub-graph* associated with node 2 (*left*) and node 4 (*right*) from the example in (**a**). To calculate the *sub-graph* entropy, we normalize the edge connection. These *sub-graphs* consist of 4-nodes. As the weighted edge between them are normalized, they sum up to 1. (**d**) An example of *sub-graph* containing edge 1–2 from the example in (**a**). To calculate the *sub-graph* entropy, we normalize the edge weights. This *sub-graph* consists of 5-nodes. As the weighted edges between them are normalized, they sum up to 1.

For a graph *G* = (*V*, *E*), let two nodes be *v*_*i*_ and *v*_*j*_. The weight of the edge between two nodes *v*_*i*_, *v*_*j*_ is denoted by *e*_*ij*_. We illustrate the approach to calculate graph entropy using an example graph shown in Fig. 2.

The example graph depicted in Fig. 2(a) consists of 7-nodes and 10-edges. For simplicity assume that the edge weights are already normalized, i.e., they sum up to 1. In this scenario, we can calculate the graph entropy as follows.

- Identifying the normalized edges *q*_*i*,*j*_. Let us identify adjacency matrix *Q* such as *Q*(*i*, *j*) = *q*_*i*,*j*_
  $$Q=[\begin{array}{ccccccc}0 & 0.05 & 0 & 0 & 0.3 & 0 & 0\\ 0.05 & 0 & 0.05 & 0 & 0 & 0.1 & 0\\ 0 & 0.05 & 0 & 0.1 & 0 & 0 & 0\\ 0 & 0 & 0.1 & 0 & 0.05 & 0 & 0.1\\ 0.3 & 0 & 0 & 0.05 & 0 & 0.1 & 0.1\\ 0 & 0.1 & 0 & 0 & 0.1 & 0 & 0.05\\ 0 & 0 & 0 & 0.1 & 0.1 & 0.05 & 0\end{array}]$$
- Calculating the entropy as $H(G)=-\,{\sum }_{i,j}\,{q}_{i,j}{\mathrm{log}}_{2}({q}_{i,j})\,=$ $-\mathrm{[4}\times 0.05\times {\mathrm{log}}_{2}\mathrm{(0.05)}+5\times 0.1\times {\mathrm{log}}_{2}\mathrm{(0.1)}\,+$ $0.3\times {\mathrm{log}}_{2}\mathrm{(0.3)]}=3.0464$ bits.

In this example, a *sub-graph* is shown in Fig. 2(b). The normalized incidence matrix of this *sub-graph* is given by

$${Q^{\prime} }_{s}=[\begin{array}{ccccc}0 & 0.09 & 0 & 0 & 0.55\\ 0.09 & 0 & 0.09 & 0 & 0\\ 0 & 0.09 & 0 & 0.18 & 0\\ 0 & 0 & 0.18 & 0 & 0.09\\ 0.55 & 0 & 0 & 0.09 & 0\end{array}]$$

The entropy can be calculated as $H({G}_{s})=-\,{\sum }_{k,m}\,{q^{\prime} }_{k,m}{\mathrm{log}}_{2}({q^{\prime} }_{k,m})\,=$ $-\mathrm{[2}\times 0.09\times {\mathrm{log}}_{2}\mathrm{(0.09)}+2\times 0.18\,\times$ ${\mathrm{log}}_{2}\mathrm{(0.18)}+0.55\times {\mathrm{log}}_{2}\mathrm{(0.55)]}=1.8576$ bits. Note that, this *sub-graph* entropy is less than actual graph entropy, indicating that it contains less randomness compared to the previous graph.

*Importance* of a graph node can be thought to be dependent on the entropy of *sub-graph*s in its immediate neighborhood. In order to calculate the entropy of *sub-graph*s surrounding a node, we need to extract the structure of *sub-graph*s containing that node. After that, based on *sub-graph* complexity, we can calculate the *sub-graph* entropy. In this example, *sub-graph*s containing nodes 2 and 4, respectively, are shown in Fig. 2(c). The normalized incidence matrix of the *sub-graph* related to node 2 is given by

$${Q^{\prime} }_{{v}_{2}}=[\begin{array}{cccc}0 & 0.25 & 0 & 0\\ 0.25 & 0 & 0.25 & 0.5\\ 0 & 0.25 & 0 & 0\\ 0 & 0.5 & 0 & 0\end{array}]$$

The entropy of node 2 is given by = $-\mathrm{[0.5}\times {\mathrm{log}}_{2}\mathrm{(0.5)}+0.25\times {\mathrm{log}}_{2}\mathrm{(0.25)}+0.25\times {\mathrm{log}}_{2}\mathrm{(0.25)]}=1.500$ bits.

On the other hand, The normalized incidence matrix of the *sub-graph* related to node 4 is given by

$${Q^{\prime} }_{{v}_{4}}=[\begin{array}{cccc}0 & 0.4 & 0 & 0\\ 0.4 & 0 & 0.2 & 0.4\\ 0 & 0.2 & 0 & 0\\ 0 & 0.4 & 0 & 0\end{array}]$$

The entropy of node is 4 given by = $-\mathrm{[0.4}\times {\mathrm{log}}_{2}\mathrm{(0.4)}+0.4\times {\mathrm{log}}_{2}\mathrm{(0.4)}+0.2\times {\mathrm{log}}_{2}\mathrm{(0.2)}]=1.5230$ bits. Note that, although the degree of node 2 and 4 are the same, their entropy values are different. The node entropy proposed in this paper is different from vertex *strength*^40^ where the *strength* of vertex is calculated as sum of edge weights associated with the vertex.

In this example, a *sub-graph* containing edge 1 − 2 is shown in Fig. 2(d).

The normalized incidence matrix of this *sub-graph* is given by

$${Q^{\prime} }_{{e}_{12}}=[\begin{array}{ccccc}0 & 0.1 & 0 & 0.6 & 0\\ 0.1 & 0 & 0.1 & 0 & 0.2\\ 0 & 0.1 & 0 & 0.0 & 0\\ 0.6 & 0 & 0 & 0 & 0\\ 0 & 0.2 & 0 & 0 & 0\end{array}]$$

The entropy can be calculated as = $-\mathrm{[0.6}\times {\mathrm{log}}_{2}\mathrm{(0.6)}+0.2\times {\mathrm{log}}_{2}\mathrm{(0.2)}+0.1\times {\mathrm{log}}_{2}\mathrm{(0.1)}+0.1\,\times$ ${\mathrm{log}}_{2}\mathrm{(0.1)]}=1.5710$ bits. This entropy is more than the node entropy calculated before, implying the edge contains more information.

#### Average Entropy from a Group of Graphs

In order to infer entropy information from a group of graphs, their sample average can be calculated. In this case, entropy values for each node and edge for each graph are calculated and the average value across all graphs is computed. This average entropy acts as an unbiased estimator for the group. For proof, see Subsection S.10 in the Supplementary Information.

### Importance of Nodes and Edges

#### Ranking of Regions

The *importance* of nodes can be described by the complexity it contains. If the *sub-graph* entropy is able to explain most complexity of the network, then those *sub-graph*s are more important. In other words, if node entropy is higher, then that node is more important in the whole network. Hence, we rank the regions based on node entropy $H({G}_{{v}_{i}})$. From a group of graphs, node entropy is calculated for each node for every graph in the group. Then we calculate the average of each node entropy for the whole group and rank the vertices based on the group averaged node entropy. The algorithm to rank the regions based on node entropy is given in Algorithm 1. The ranking pipeline is also illustrated in Fig. S1 in Supplementary Information.

**Algorithm 1:**
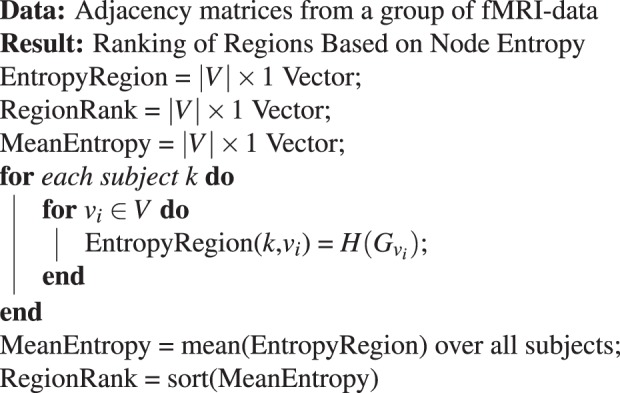
Ranking of Regions.

This scheme can be seen as maximizing mutual information between *sub-graph* and the whole graph. We provide a proof in the Supplementary Information Subsection S.11.

We use the node entropy to rank the regions of brain which are most *important* for different conditions (emotion task, gambling task, no-task) using Algorithm 1. The result of the ranking process for emotion task is shown in Table 1 and Fig. S2 in Supplementary Information. The regions of *importance* were consistent almost for every *state*, i.e., the regions that carried the most entropy did not change between task *vs*. no-task *states*.

**Table 1:** *Left*: Top regions employed for emotion task according to Algorithm 1.

Region	Entropy	Edge		Entropy
Temporal Pole - R	5.9912	Parstriangularis - L	Temporalpole - R	6.7815
Temporal Pole - L	5.8128	Insula - L	Temporalpole - R	6.7339
Parstriangularis - L	5.6191	Temporalpole - L	Temporalpole - R	6.7341
Insula - R	5.6073	Parstriangularis - L	Insula - R	6.697
Parstriangularis - L	5.5606	Temporalpole - L	Insula - R	6.6936
Entorhinal - R	5.332	Parsopercularis - L	Temporalpole - R	6.6602
Insula - L	5.3212	Temporalpole - L	Parstriangularis - R	6.6488
Amygdala - R	5.2477	Inferiortemporal - L	Temporalpole - R	6.6433
Parsopercularis - R	5.2355	Temporalpole - L	Parsopercularis - R	6.6427
Inferiortemporal - R	5.23	Parstriangularis - L	Entorhinal - R	6.6381
Parsopercularis - L	5.0683	Entorhinal - L	Temporalpole - R	6.6161
Inferiortemporal - L	5.067	Putamen - R	Temporalpole - L	6.611
Putamen - R	5.0523	Temporalpole - L	Entorhinal - R	6.6097
Parsorbitalis - R	5.0105	Superiortemporal - L	Temporalpole - R	6.6034
Superiortemporal - R	4.9332	Insula - L	Parstriangularis - R	6.6027
Rostralmiddlefrontal - R	4.924	Amygdala - R	Parstriangularis - L	6.6012
Entorhinal - L	4.7726	Fusiform - L	Temporalpole - R	6.5989
Frontalpole - R	4.7241	Temporalpole - L	Inferiortemporal - R	6.5952
Medialorbitofrontal - R	4.7214	Parstriangularis - L	Parstriangularis - R	6.5944
Fusiform - R	4.7196	Temporalpole - L	Rostralmiddlefrontal - R	6.5925
Rostralanteriorcingulate - R	4.7017	Parstriangularis - L	Parsopercularis - R	6.5889
Fusiform - L	4.6306	Parstriangularis - L	Inferiortemporal - R	6.5767
Superiortemporal - L	4.5888	Temporalpole - L	Parsorbitalis - R	6.5754
Caudalanteriorcingulate - L	4.4629	Putamen - R	Parstriangularis - L	6.5752
Bankssts - L	4.3983	Bankssts - L	Temporalpole - R	6.5649

*Right*: Top edges associated with the emotion task according to Algorithm 2.

#### Ranking of Edges

In this experiment, the edges are ranked based on edge entropy $H({G}_{{e}_{ij}})$. As before, the edge entropy of each edge for every graph is calculated from a group of graphs. Then we compute the average of each edge entropy for the whole group and rank the nodes based on the group averaged edge entropy. The algorithm to rank the edges based on edge entropy is given in Algorithm 2.

**Algorithm 2:**
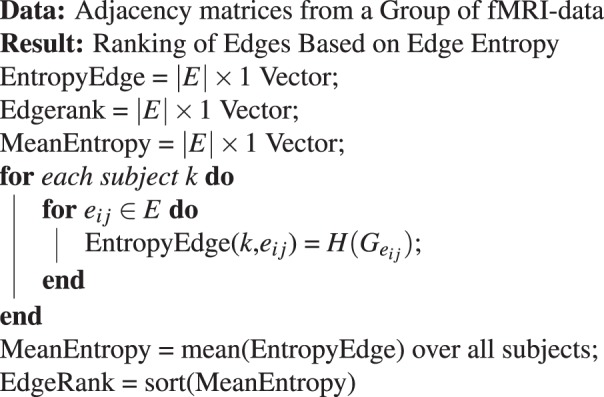
Ranking of Edges.

Edge entropy is then used to rank the functional edges of brain according to the *importance* of priority for different conditions (emotion task, gambling task, no-task) using Algorithm 2. The result of this ranking process for emotion task is shown in Table 1. The top-100 active edges are shown in Fig. S3 in Supplementary Information. The *importance* of priority edges was consistent for every *state*. In all the *states*, the most *important* edges are those criss-crossing two hemispheres. Also, the edges are mostly concentrated in the frontal regions of the brain. This is also consistent with the nodes found in regional ranking for each separate condition.

#### Ranking based on Differential Entropy

Between two groups of tasks (or task *vs*. no-task conditions), if the communication pattern among brain regions change, then the change in pattern can be captured using the above mentioned ranking procedure. In this scenario, the regions or links with the most change in entropies between two groups play a significant role in discriminating the two classes. Suppose, for region *v*_*i*_, the conditional entropy for subjects belonging to group *G*1 (where *G*1 ∈ {Emotion, Gambling}) is given by *H*_*G*1_(*v*_*i*_) and for group *G*2 (where *G*2 ∈ {No-task, Other Task}), *H*_*G*2_(*v*_*i*_). The difference between these two values would encompass the change in graph entropies between two groups of subjects for region *i*. We calculate the change in entropy (defined differential entropy) as |*H*_*G*1_(*v*_*i*_) − *H*_*G*2_(*v*_*i*_)| where |*x*| is the absolute value of *x*. Then we rank them based on decreasing value. The results from our experiment show empirically that this ranking can capture the significant distinguishing regions between two groups. The same argument and ranking procedure can be applied to edges as well. The algorithm is described in Algorithm 3.

**Algorithm 3:**
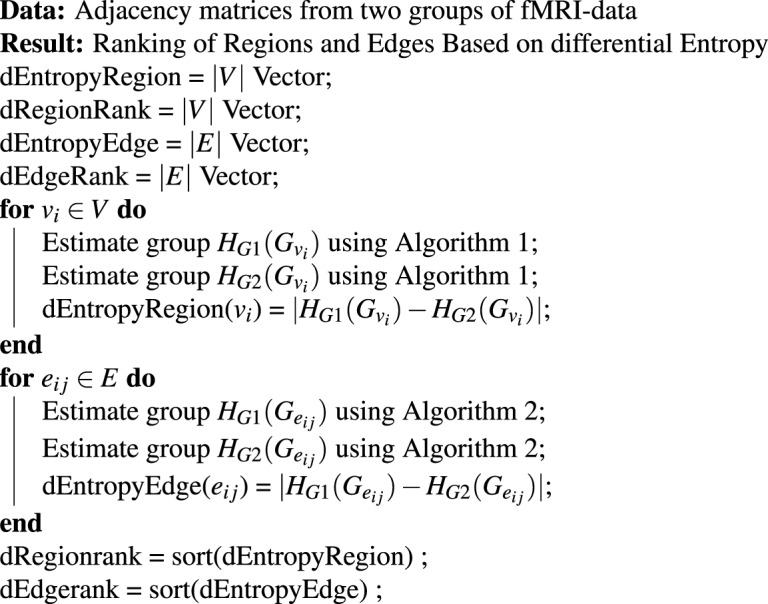
Ranking of Regions and Edges for Two Groups based on differential entropy.

**Figure 3:**
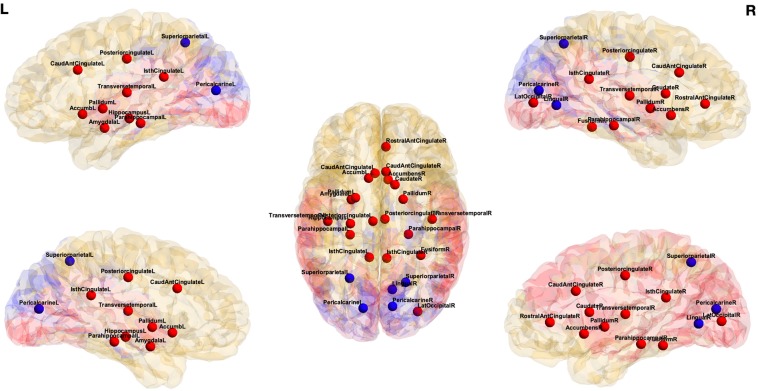
Visualization of *important* regions that have highest differential entropy between two *states* for emotion task *vs*. no-task. *Red:* regions that have higher node entropy during emotion task, *blue:* regions that have higher node entropy during no-task.

There are regions that have maximum change of entropy between two *states*. Although, these regions may not be among the most complex regions, they provide the maximum change of entropy between two *states*. We extract the regions that are *important* from the perspective of change of information in Table 2 for different tasks. The corresponding regions of interest for emotion *vs*. no-task are shown in Fig. 3. In addition, the regions of interest for gambling *vs*. no-task and emotion *vs*. gambling are shown in Figs. S4 and S5, respectively (Supplementary Information).

**Table 2:** Ranking of *important* regions that have differences in entropy between two tasks.

Emotion *vs*. No-task		Gambling *vs*. No-task		Emotion *vs*. Gambling	
Regions	Diff. Entropy	Regions	Diff. Entropy	Regions	Diff. Entropy
Pericalcarine - L	0.1347	Accumbens - L	0.1355	Hippocampus - L	0.5736
Superiorparietal - L	0.1343	Pallidum - L	0.1209	Cuneus - L	0.5039
Fusiform - R	0.1212	Caudate - R	0.118	Pericalcarine - L	0.4924
Pallidum - R	0.1068	Lingual - L	0.1138	Pallidum - R	0.4222
Superiorparietal - R	0.1055	Accumbens - R	0.1055	Precuneus - L	0.4031
Amygdala - L	0.1008	Precentral - R	0.1028	Fusiform - R	0.3810
Caudate - R	0.0992	Pericalcarine - L	0.1005	Parahippocampal - R	0.3675
Pericalcarine - R	0.0981	Postcentral - L	0.0993	Putamen - L	0.3640
Hippocampus - L	0.0969	Superiorfrontal - R	0.0985	Caudalanteriorcingulate - L	0.3519
Accumbens - L	0.0964	Transversetemporal - R	0.0976	Brain Stem	0.3468
Transversetemporal - R	0.0961	Amygdala - R	0.0967	Bankssts - L	0.3370
Caudalanteriorcingulate - R	0.0912	Posteriorcingulate - R	0.0965	Supramarginal - R	0.3362
Parahippocampal - L	0.0880	Postcentral - R	0.0878	Superiorparietal - R	0.3255
Rostralanteriorcingulate - R	0.0784	Pericalcarine - R	0.0872	Pericalcarine - R	0.3080
Isthmuscingulate - L	0.0767	Brain Stem	0.0863	Left Pallidum	0.3069
Parahippocampal - R	0.0762	Precentral - L	0.0788	Putamen - R	0.3049
Pallidum - L	0.0757	Parahippocampal - L	0.0771	Lateraloccipital - R	0.2851
Lateraloccipital - R	0.0714	Lateralorbitofrontal - L	0.0739	Accumbens - R	0.2810
Posteriorcingulate - R	0.0708	Inferiorparietal - L	0.0734	Hippocampus - R	0.2783
Transversetemporal - L	0.0648	Parsopercularis - L	0.0720	Transversetemporal - L	0.2604
Posteriorcingulate - L	0.0635	Cerebellum Cortex - R	0.0700	Transversetemporal - R	0.2584
Lingual - R	0.0566	Caudate - L	0.0696	Parsorbitalis - R	0.2538
Caudalanteriorcingulate - L	0.0550	Frontalpole - L	0.0684	Cerebellum Cortex - L	0.2373
Accumbens - R	0.0521	Lateralorbitofrontal - R	0.0667	Superiorfrontal - L	0.2303
Isthmuscingulate - R	0.0519	Amygdala - L	0.0658	Rostralmiddlefrontal - R	0.2224

The regions with significant change in entropy values are ranked among top 15 regions.

**Figure 4:**
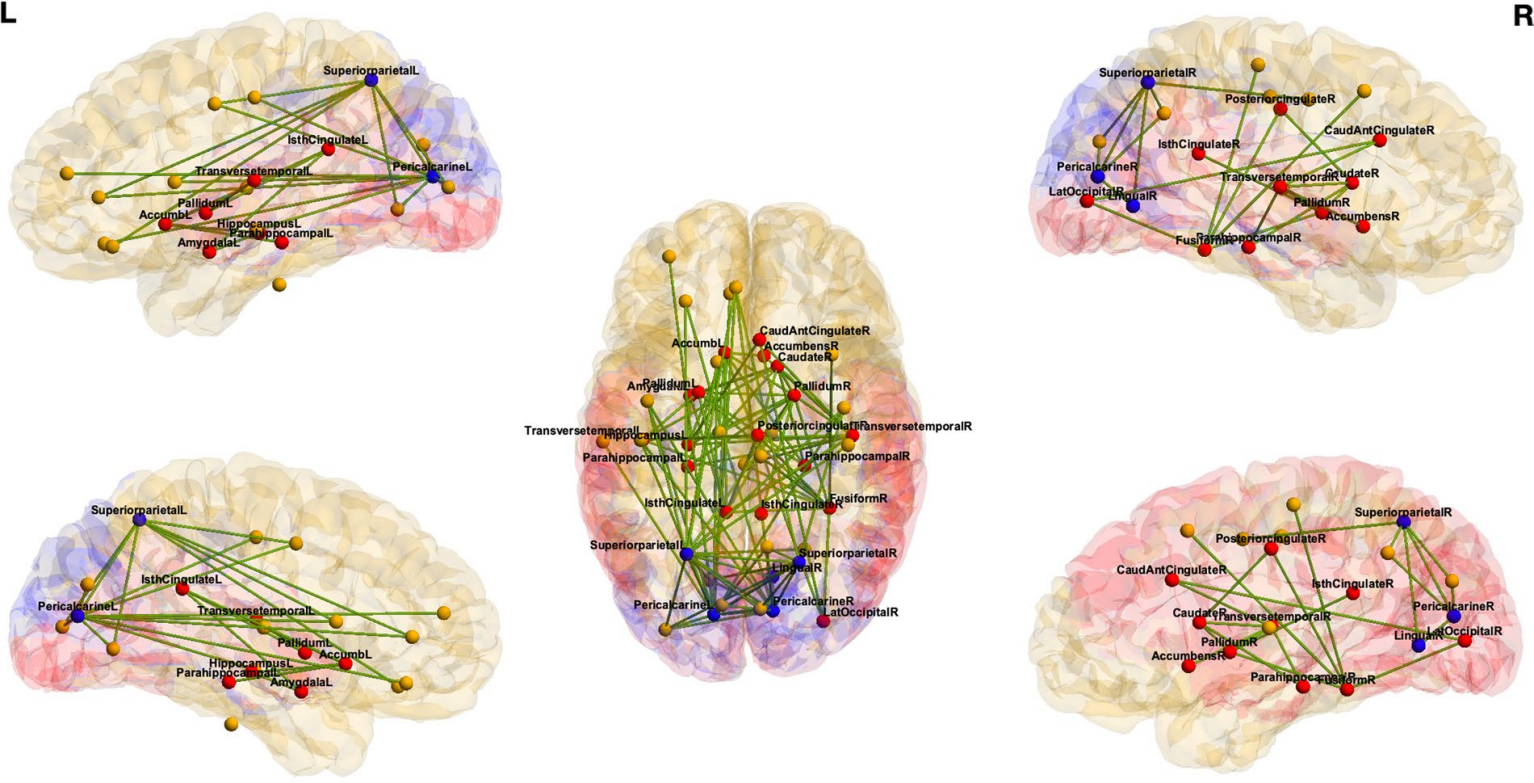
Visualization of *important* edges that have highest differential entropy between two *states* for emotion task *vs*. no-task. *Red:* regions that have higher node entropy during emotion task, *blue:* regions that have higher node entropy during no-task, *yellow:* regions that are not significant based on node entropy.

The change in ranking for emotion *vs*. no-task was the highest for fusiform cortex in the right hemisphere. For emotion *vs*. gambling task, the regions with maximum change in ranking for individual tasks are: left hemisphere banks of the superior temporal sulcus, left caudal anterior cingulate and right fusiform cortex. In order to facilitate the visualization of edge ranking procedure, the top ranked edges are overlaid on a brain template. Following group ranking procedure based on edge entropy, this process extracts top edges from (a) emotion *vs*. no-task (Fig. 4), (b) gambling *vs*. no-task (Fig. S6 in Supplementary Information) (c) emotion *vs*. gambling (Fig. S7 in Supplementary Information). These edges are also listed in Supplementary Information Tables S1 and S2. Here the top 100 edges for each group are identified. A close inspection of the results reveals several observations. First, group ranking procedure reveals edges that are distributed throughout the whole brain and some of them criss-cross the hemispheres. Second, differential entropy elevates the edges that belong to frontal-parietal and frontal-subcortical areas, *e*.*g*., frontal lobe, parietal lobe, temporal lobe, cingulate gyrus, limbic system, striatum, thalamus, stem, and amygdala.

### Performance of Classifying Two Brain States

The leave-one-out classification performance using top-25 region and significant edge entropies are shown in Table 3. The classification performance is compared with state-of-the-art network metrics for nodes. In addition, the classification performance for edges is also compared to NBS measures. A number of classifiers were tested, *e*.*g*., support vector machine (SVM), random forest, naive Bayes, and logistic regression. All the classifiers perform similarly with respect to the features. Therefore, the results from support vector machine with radial basis function are presented for illustration. The hyperparameters for the classifiers were tuned using in-fold validation. The support vector machine classifier with a radial basis function kernel and node entropy features performs better for classifying two *states* with highest accuracy, specificity and sensitivity between node and edge based features separately.

**Table 3:** Classification performance for three classification tasks.

Region Performance	Emotion *vs*. No-task				Gambling *vs*. No-task				Emotion *vs*. Gambling			
	# features	Accuracy	Specificity	Sensitivity	# features	Accuracy	Specificity	Sensitivity	# features	Accuracy	Specificity	Sensitivity
Node Entropy (proposed)	25	**0.96**	**0.94**	**0.98**	**25**	**0.97**	**0.96**	**0.97**	**25**	**0.94**	**0.97**	**0.91**
Degree^24^	85	0.85	0.90	0.82	85	0.92	0.94	0.89	85	0.91	0.90	0.93
Eigenvector^25^	85	0.90	0.87	0.93	85	0.94	**0.96**	**0.94**	**85**	**0.94**	**0.92**	**0.95**
Betweenness^26^	85	0.82	0.87	0.80	85	0.89	0.91	0.89	85	0.86	0.88	0.84
Leverage^22^	85	0.83	0.85	0.81	85	0.87	0.83	0.91	85	0.83	0.87	0.80
Tensor based^89^	1280	0.91	0.93	0.89	1280	0.92	0.88	0.94	1280	0.90	0.85	**0.95**
**Edge Performance**	**# features**	**Accuracy**	**Specificity**	**Sensitivity**	**# features**	**Accuracy**	**Specificity**	**Sensitivity**	**# features**	**Accuracy**	**Specificity**	**Sensitivity**
Edge Entropy (proposed)	102	**0.91**	**0.94**	**0.87**	**118**	**0.96**	**0.96**	**0.95**	**83**	**0.94**	**0.94**	**0.95**
NBS^28^	536	0.86	0.81	0.90	1527	0.8	0.86	0.75	1496	0.88	0.92	0.85
**Sub-graph Performance**	**# features**	**Accuracy**	**Specificity**	**Sensitivity**	**# features**	**Accuracy**	**Specificity**	**Sensitivity**	**# features**	**Accuracy**	**Specificity**	**Sensitivity**
Sub-graph - 1 (Intersection)	77	0.95	0.94	0.96	89	0.94	0.96	0.93	42	0.91	0.93	0.90
Sub-graph - 2 (Union)	127	**0.97**	**0.95**	**0.98**	**143**	**0.97**	**0.96**	**0.97**	**108**	**0.95**	**0.97**	**0.94**

Performance of node entropy is compared with other region based centrality measures. Performance of edge entropy is compared with NBS. In addition, classification performance for sub-graph containing intersection and union of top regions and edges are demonstrated. Sub-graph - 1 (intersection) contains top-25 regions and significant edges where nodes belong to top-25 regions. Sub-graph - 2 (union) contains top-25 regions and significant edges.

Intersection and Union Sub-Graphs: Two *sub-graphs* are created from the intersection and union of top regions and edges to compute *sub-graph* entropies for different groups. The *intersection sub-graph* contains subset of edges associated with the nodes of the top-25 regions. The *union sub-graph* contains top-25 regions and significant edges. The node and edge entropies associated with *union* and *intersection sub-graphs* are also used for classification. These results are summarized in Table 3.

When we utilize the regional centrality measures based on the regions of Table 2 to classify task *vs*. no-task *states* or emotion *vs*. gambling *states*, the classifier achieves very good area under the curve (AUC) values (shown in Fig. S8 in Supplementary Information). Compared to other centrality measurements, the proposed centrality achieves better prediction consistently for the whole range of receiver operating characteristics (ROC). Using edge entropies, the proposed classifier achieves very good mean AUC values as shown in Fig. S9 in Supplementary Information.

### Statistical Analysis of Results

#### Significance of Regions and Edges

The statistical significance of the top ranked regions that have highest change in node entropy is investigated using *nonparametric permutation t-test* separately on each highly ranked regions. For emotion *vs*. no-task, out of the 25 regions shown in Table 2, top 11 have significant change in node entropy. For gambling *vs*. no-task, top 15 regions have significant change in node entropy. The same procedure, using t-test, is also carried out using other four centrality measures, i.e., *degree*, *betweenness*, *eigenvector* and *leverage* centrality. The significant regions found using the other centrality measures are shown in Tables S3 and S4 in Supplementary Information. Node entropy measure is always able to extract the regions found to be significant by other measures. In addition, it finds some other *important* regions not found by the state-of-the-art centrality measures. For emotion task, the regions shown to be significant by node entropy, but not by other measures, include: left hippocampus, left amygdala, left accumbens, right caudate, right pallidum and right transversetemporal. Similarly, for gambling task, the regions shown to be significant by node entropy, but not by other measures, include: left pericalcarine, right pericalcarine, right postcentral and right transversetemporal.

For edges, *nonparametric permutation t-test* is carried out using edge entropy values on all edges, and the statistically significant edges are found using *p* = 0.05 with Bonferroni correction. The sub-network containing the significant edges are all top ranked edges from Algorithm 3. The number of edges, that had significant change in edge entropy values correspond to 102, 118 and 83, respectively, for emotion *vs*. no-task, gambling *vs*. no-task and emotion *vs*. gambling. The sub-networks containing the edges are shown in Fig. 4 for emotion *vs*. no-task.

#### Stability of Top Regions and Edges

We use a rigorous leave-one-out technique to rank regions and edges in order to understand the stability of our method^41–44^. We run the proposed algorithm (Algorithm 3) 475 times, each time leaving one subject out and ranking the regions and edges based on Algorithm 3. We find that, the top regions and edges obtained from this leave-one-out method are very stable as shown by their histograms. For emotion task, top 21 regions (from Table 2) were ranked among top 25 regions 475 times, the rest four regions came up 474, 470, 447 and 412 times, respectively. For gambling task, top 20 regions (from Table 2) came up 475 time, the rest five regions were ranked *important* 474, 470, 465, 445 and 438 times, respectively. For differentiating emotion *vs*. gambling, top 21 regions (from Table 2) were ranked higher 475 time, the rest five regions came up 470, 375, 360, 325 times, respectively. Out of the significant edges for three tasks, 75%, 85%, 80% edges, respectively, came up 475 times. The number of occurrences of the regions (and edges) among top-25 (and significant edges, respectively) are illustrated in Figs S10 and S11 (Supplementary Information), respectively. The histogram for each case is quite flat signifying that important regions and edges were similar across most subjects. This indicates a consistent group-level behavior for classification, i.e., same features are being used for classifying two *states*.

#### Quantifying Classification Significance

To further establish that the results are better than chance, we perform permutation tests. Performing permutation test involves computing a trivial baseline using permuted labels, i.e., the accuracy produced if there was “no signal” between the features and label. Then we determined if our learned model performed significantly better than the baseline. Here, for each dataset (emotion *vs*. no-task, gambling *vs*. no-task, emotion *vs*. gambling), we performed 1000 iterations: each time, we randomly permuted the subject labels to effectively remove any relationship between the input features and the label, then we trained a model on the training subset of this set and tested it on the remaining subset. Fig. S12 shows the distributions of accuracy scores for the three datasets. In each case, we see that there is a significant difference between the centers of the distributions and the accuracy obtained by node entropy (*p* = 4.8213 × 10^−8^, 7.7689 × 10^−11^, 9.8659 × 10^−10^, respectively, for three tasks). The same conclusion holds for edge entropy. In addition to the permutation tests, we use a binomial test to compare the leave-one-out classification accuracies (using node and edge entropy) to baseline accuracies, to determine if each learner is significantly better than previous state-of-the-art classifiers. Node entropy performs significantly better than the next best method (tensor based) for classifying emotion *vs*. no-task with *p* = 7.9637 × 10^−7^. In addition, it is significantly better than eigenvector centrality for classifying gambling *vs*. no-task (*p* = 7.3483 × 10^−4^). Edge entropy is also better than NBS based methods for classification with *p* = 4.0653 × 10^−4^, 1.5673 × 10^−15^, 5.8537 × 10^−6^, respectively, for three classification tasks. The highest classification performance is achieved using node and edge entropy features associated with the *union sub-graph*.

#### Comparison of Node Entropy based Importance with Other Measures

**Figure 5:**
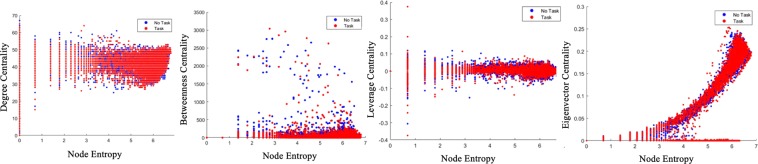
Comparing centrality measures for *emotion* task. *Left* to *right*: Information-theoretic centrality *vs*. *degree*, *betweenness*, *leverage*, *eigenvector* centrality. For each region the measures were plotted and overlaid for all subjects. Number of points for task and no-task are same.

To understand the relationship between the proposed measure and other well-known centrality measures in fMRI literature, we use a scatter plot of the node entropy values for both task and no-task conditions with other centrality measures in Fig. 5 for emotion task. The gambling task follows similar pattern and has not been shown here. In addition, we calculate mean correlation values of centrality measures for a group of graphs (both simulated and real world) in Table 4. The simulated graphs are first constructed using 85 nodes and edges following a uniform distribution (0–1). Next, the graphs are made sparse similar to the sparsity of real networks. For each graph, node entropies are measured and correlated with other centrality measures. Then the average and standard deviation values of correlation are calculated. Correlation values are similarly calculated for the data from emotion and gambling tasks. The scatter plot and the table indicate that our proposed centrality measure has very low correlation values with *degree*, *betweenness* and *leverage* centrality although it has a somewhat high correlation with *eigenvector* centrality. This implies that graph entropy provides a different dimension of *importance* in comparison with *degree*, *betweenness* and *leverage*, and provides somewhat similar information with *eigenvector* centrality.

**Table 4:** Comparison of correlation values between graph entropy and other centralities.

	*Degree vs*. Node Entropy	*Betweenness vs*. Node Entropy	*Leverage vs*. Node Entropy	*Eigenvector vs*. Node Entropy
Simulated	0.3899 ± 0.0928	0.6865 ± 0.0451	0.36450 ± 0.0883	0.9412 ± 0.0120
Emotion	0.2478 ± 0.1867	0.2672 ± 0.0689	0.0343 ± 0.0430	0.5912 ± 0.0499
Gambling	0.2475 ± 0.1782	0.2666 ± 0.0686	0.0346 ± 0.0410	0.5876 ± 0.0487

We also performed GLM analysis of the two tasks. Based on the value of regression coefficients, we ranked the regions associated with each task separately. The ranked regions are shown in Table 5.

**Table 5:** Regions with high regression coefficients from generalized linear model analysis.

Emotion *vs*. No-task				Gambling *vs*. No-Task			
Entropy Based		GLM Based		Entropy Based		GLM Based	
Regions	Entropy	Regions	Reg. Coeff	Regions	Entropy	Regions	Reg. Coeff
Pericalcarine - L	0.1347	**Lateraloccipital - R**	1	Accumbens - L	0.1355	Caudalanteriorcingulate - R	1
Superiorparietal - L	0.1343	Lateraloccipital - L	0.9727	Cuneus - L	0.1209	Caudalanteriorcingulate - L	0.9749
**Fusiform - R**	0.1212	**Fusiform - R**	0.9492	**Caudate - R**	0.1180	**Superiorfrontal - R**	0.9568
Pallidum - R	0.1068	Amygdala - R	0.8540	Lingual - L	0.1138	Superiorfrontal - L	0.9359
Superiorparietal - R	0.1055	Fusiform - L	0.8443	Accumbens - R	0.1055	Rostralanteriorcingulate - R	0.9172
**Amygdala - L**	0.1008	**Pericalcarine - R**	0.7019	Precentral - R	0.1028	**Caudate - L**	0.8908
Caudate - R	0.0992	**Amygdala - L**	0.6902	Pericalcarine - L	0.1005	Rostralmiddlefrontal - L	0.8893
**Pericalcarine - R**	0.0981	Lingual - L	0.6223	Postcentral - L	0.0993	Rostralanteriorcingulate - L	0.8832
Hippocampus - L	0.0969	**Caudalanteriorcingulate - L**	0.6091	**Superiorfrontal - R**	0.0985	Thalamus - L	0.8789
Accumbens - L	0.0964	Temporalpole - L	0.5611	Transversetemporal - R	0.0976	Insula - L	0.8769
Transversetemporal - R	0.0961	Lingual - R	0.5485	**Amygdala - R**	0.0967	**Amygdala - R**	0.8719
**Caudalanteriorcingulate - R**	0.0912	**Caudalanteriorcingulate - R**	0.5450	Posteriorcingulate - R	0.0965	Insula - R	0.8708
Parahippocampal - L	0.0880	Superiorparietal - L	0.5385	Postcentral - R	0.0878	Thalamus - R	0.8689
Rostralanteriorcingulate - R	0.0784	Bankssts - R	0.5156	Pericalcarine - R	0.0872	**Caudate - R**	0.8624
Isthmuscingulate - L	0.0767	Rostralanteriorcingulate - L	0.5080	**Brain Stem**	0.0863	Hippocampus - L	0.8502
Parahippocampal - R	0.0762	Pericalcarine - L	0.4778	Precentral - L	0.0788	Rostralmiddlefrontal - R	0.8496
Pallidum - L	0.0757	Entorhinal - L	0.4725	Parahippocampal - L	0.0771	Parsopercularis - R	0.8494
**Lateraloccipital - R**	0.0714	Supramarginal - R	0.4621	Lateralorbitofrontal - L	0.0739	**Parsopercularis - L**	0.8460
**Posteriorcingulate - R**	0.0708	Superiorparietal - R	0.4389	Inferiorparietal - L	0.0734	**Brain Stem**	0.8387
Transversetemporal - L	0.0648	Rostralanteriorcingulate - R	0.4320	**Parsopercularis - L**	0.0720	Parstriangularis - L	0.8360
Posteriorcingulate - L	0.0635	Supramarginal - L	0.4300	Cerebellum Cortex - R	0.0700	Parstriangularis - R	0.8341
Lingual - R	0.0566	**Posteriorcingulate - R**	0.4274	**Caudate - L**	0.0696	**Amygdala - L**	0.8316
**Caudalanteriorcingulate - L**	0.0550	Precentral - L	0.4140	Frontalpole - L	0.0684	Hippocampus - R	0.8278
Accumbens - R	0.0521	Postcentral - L	0.4131	Lateralorbitofrontal - R	0.0667	Caudalmiddlefrontal - L	0.8276
Isthmuscingulate - R	0.0519	Brain Stem	0.4047	**Amygdala - L**	0.0658	Caudalmiddlefrontal - L	0.8244

The regression coefficients are divided by the highest coefficient. Ranked regions from entropy models are also shown for comparison. The common regions are in bold.

#### Comparison of Graph Entropies between Two States

The total graph entropy values between *states* corresponding to two conditions (task *vs*. no-task time points or task 1 *vs*. task 2) are also compared. After the calculation of two types of graph entropies for each subject, a one-sided t-test is carried out to understand if the two *states* were significantly different. Graph entropy based *p-values* for *functional connectivity states* are shown in Table 6. All the changes were statistically significant (*p* < 0.05). The corresponding group mean entropy values are also plotted and compared for two different *states* (task *vs*. no-task conditions or others) in Fig. S14 in Supplementary Information. We use standard box plot to visualize the span of entropy values for each group. For classification between two *states*, this feature achieves greater than 0.7 area under curve (AUC) for classification for all cases. The *sub-graph* entropies between two *sub-graphs* are also compared across different tasks and illustrated as box-plots in Figs S15, S16, respectively, in Supplementary Information.

**Table 6:** Graph entropy difference between two conditions for brain network.

	Emotion Task *vs*. No-task	Gambling Task *vs*. No-task	Gambling Task *vs*. Emotion Task
	*p-value* (Effect Size)	*p-value* (Effect Size)	*p-value* (Effect Size)
Graph Entropy	2.1344e-31 (0.57)	1.8636e-21 (0.45)	1.0721e-09 (0.29)

## Discussion

The important regions and edges extracted using only one condition are similar across all subjects. They are concentrated mainly in the frontal part of the brain. There are no significant differences between important regions and edges for different conditions. These regions and their connectivities are commonly used in brain to transfer information during task. Many of the significant regions are in anterior cingulate gyrus, ventromedial frontal cortex, and inferior parietal brain regions. These regions are consistent with the previous works by Cole *et al*.^45^, Tomasi *et al*.^46^, Zuo *et al*.^18^. We provide theoretical justifications in Supplementary Information Subsections S.10, S.11 and S.12 for using edge strength and average graph entropy as a measure of group-level behavior of *states* and show that maximizing *sub-graph* entropy leads to maximizing mutual information between a sub-structure and whole graph. Some of the regions extracted using one condition consist of some small and noisy regions like left temporal pole and right temporal pole. These regions are ranked lower when using differential entropy. Generally, smaller and noisier regions will not rank higher when differential entropy is used.

### Emotion Task

Our definition of important regions between two different conditions based on change of information flow could also extract regions most responsible for the tasks. We also identify a number of useful brain functional areas that are activated mainly during emotion tasks as significant regions between task *vs*. no-task networks. These areas are amygdala, caudate region, fusiform, striatum, and basal ganglia. Fusiform gyrus has been identified as one of the main regions for face information processing in Mccarthy *et al*.^47^. This region is also identified as one of the main regions for face emotion processing^48,49^. We find this region among top-5 regions in our ranking. Pallidum, part of basal ganglia, is also a very important region in terms of emotion processing. Nucleus accumbens area (both right and left hemisphere) is also identified as a significant region. Neuclus accumbens has been shown to be an important area for emotional processing in^50–52^. Specially, Floresco *et al*.^52^ hypothesize it to be an intermediary region regulating cognition and action. Areas from anterior cingulate cortex have been related to cognition and emotion^53^. Moreover, regions from anterior cingulate cortex (ACC) are related to intelligent behavior, i.e., emotional self-control, focused problem solving, error recognition, and adaptive response to changing conditions^54^. Also, Etkin *et al*.^55^ showed its involvement in negative emotional stimuli^55^. We find hippocampal areas to have significant changes during emotion both for emotion *vs*. no-task and emotion *vs*. gambling. Hippocampus has been correlated with emotional responses and acts in conjunction with amygdala for processing of emotional situations. The amygdala and hippocampal areas, two medial temporal lobe structures, are linked to two independent memory systems, each with their unique characteristic functions, respectively. The situation where a person faces emotional stimuli, the two regions interact to give rise to specific responses. Specifically, amygdala can have effect on both the formation and storing of memories that depend on hippocampal activation^56^. The hippocampus area is associated with the amygdala response by forming episodic representations of the emotional stimuli. Although these regions are independent with respect to memory organization, they act in concert when emotion stimuli meets memory representations^56^.

The emotion task based on visual face information has a great effect on the regions from visual cortex specifically V1 areas. Calcarine sulcus areas from both right and left hemispheres have the most change in information flow in case of regions and edges. Areas from parietal lobule are also identified as important regions to explain the functional network. These regions may have been prominent as they have been shown to be responsible for processing higher order facial features^57^. One of the surprising finding is the ranking of caudate neucleus as an important region during the task. Caudate has generally been correlated with emotional processing but not with respect to the reaction to the preference of face pictures^58,59^. It has also been identified as neural correlate for emotion based heart rate variability^60^. Hence, apart from main hub locations for angry or fearful emotions, brains of the subjects may also try to process multiple dimensions of the visual stimuli. The edges extracted as important edges also support the regional involvement as most of the regions in the edges are similar as in Table 2. All the regions and edges have *p-value* < 0.05 indicating that they are statistically significant as well.

### Gambling Task

The regions that have significant change in information belong to the reward circuitry of brain. Specifically regions from orbitofrontal^61^, limbic system (amygdala, hippocampal) and basal ganglia neucleus (pallidum and striatum area caudate) were seen to have most change in entropies between gambling *vs*. no-tasks. One other area that has been shown to be involved from the proposed ranking method is neucleus accumbens. Knutson *et al*. have showed that activation in nucleus accumbens is prominent in people performing a gambling task. However, it is conjectured that this activity is associated with anticipation of reward prediction. This further reinforces the efficacy of differential entropy for ranking process using gambling task without the monetary reward^62,63^. Moreover, reward processing is also correlated with reward-related functional activation in the nucleus accumbens^64^. In case of reward prediction, a behavior employed by the gambling task, significant activity is seen in the lateral orbitofrontal cortex and the striatum^65^. Basal ganglia region striatum is seen to be related to differentiating rewards from non-rewards^66^. Human brain limbic system is associated with neural responses for reward prediction^67^. Especially the difference between the actual gain and expected gain are associated with a neural circuitry of the mesolimbic dopamine system^68^. Gambling task also invokes areas related to decision making, *e*.*g*., amygdala. Previous studies have shown that amygdala damage can interfere with decision-making^69^. Amygdala is critical in the neural system and it triggers somatic *states* from primary inducers that brings back emotions for a secondary event. Functional disconnectivity of the amygdala regions have been shown to impair acquisition of gambling tasks in rats. It also alters their decision making behavior^70^. Anterior cingulate cortex’s involvement in cognition and conflict monitoring is well documented. Specifically, findings have posed specific challenges, especially concerning the way it addresses the processing of errors^71^. Dorsal ACC in adults are also active making risky selections. Furthermore, reduced activity in these areas are correlated with greater risk-taking performance making risky economic choices^72^. Other studies also suggest anterior cingulate is significantly correlated with performance on the gambling task^73^ and risk anticipation^74^. In addition, we also extract significant regions from frontal lobe and parietal lobe whose entropy have changed significantly during the gambling task. As before, the top ranked edges extracted as important edges also supported the regional involvement as most of the regions in the edges are similar as shown in Table 2. All the regions and edges had *p-value* < 0.05 indicating that they are statistically significant as well.

Graph entropy values can be used as a representative metric for neural *state*. On the other hand, *sub-graph* entropy metric can be used to extract useful regions and edges that have significant differences between two *states*. Some of the regions found by *sub-graph* entropy are similar to traditional GLM (Table 5). Incorporating biologically meaningful regions, edges extracted through the differential entropy based ranking procedure also outperforms other centrality measures for classifying two *states*. In addition, the centrality information conveyed by graph entropy is different compared to *degree*, *betweenness and leverage* centrality. The scatter plots between node entropy and other centralities (Fig. 5) are flat and wide implying very little overlap in the information content. Many regions extracted through *sub-graph* entropy are different which indicates that *sub-graph* entropy conveys different information regarding *functional connectivity* compared to traditional methods.

## Methods

### Dataset

Two different task-fMRI datasets collected from 475 subjects from the Human Connectome Project (HCP) Young Adult study^6,16^ were used in this paper. The tasks chosen were emotion and gambling. These data are publicly available from the ConnectomeDB database https://db.humanconnectome.org. All data were acquired on a customized Siemens 3 T Connectome Skyra scanner with the following parameters: task-fMRI was obtained with 2 mm isotropic voxels with TR = 720 ms, TE = 33.1 ms. Here emotion processing task was carried out with two runs of 2:16 min with 176 frames per each run. Gambling task was continued for 3:12 mins with 253 frames per run for two runs^75,76^.

### Description of Task

#### Emotion

**Figure 6:**
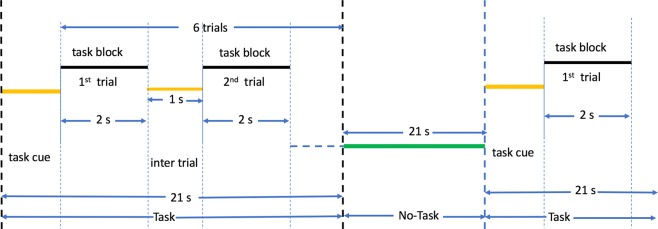
Emotion task pipeline. Each task block consists of 6 trials of emotion task paradigm following^77^ of duration 21 s. This follows by a no-task block of 21 s. There are 3 task blocks and 3 no-task blocks in total.

This task was adapted from the one developed by Hariri *et al*.^77^. Participants are presented with blocks of trials that either ask them to decide which of the two faces presented on the bottom of the screen match the face at the top of the screen, or which of two shapes presented at the bottom of the screen match the shape at the top of the screen. The faces have either an angry or fearful expression. The task format is illustrated in Fig. 6. Here 6 trials of the same task (face or shape) are repeated with the stimulus presented for 2000 ms and a 1000 ms inter-task interval (ITI). Each block is preceded by a 3000 ms task cue (“shape” or “face”) so that each block is 21 seconds long including the cue. Each of the two runs includes 3 face blocks and 3 shape blocks with 8 seconds of fixation at the end of each run. The task is described based on *WU-Minn HCP* 500 *Subjects Data Release Manual* available from https://www.humanconnectome.org/.

#### Gambling

**Figure 7:**
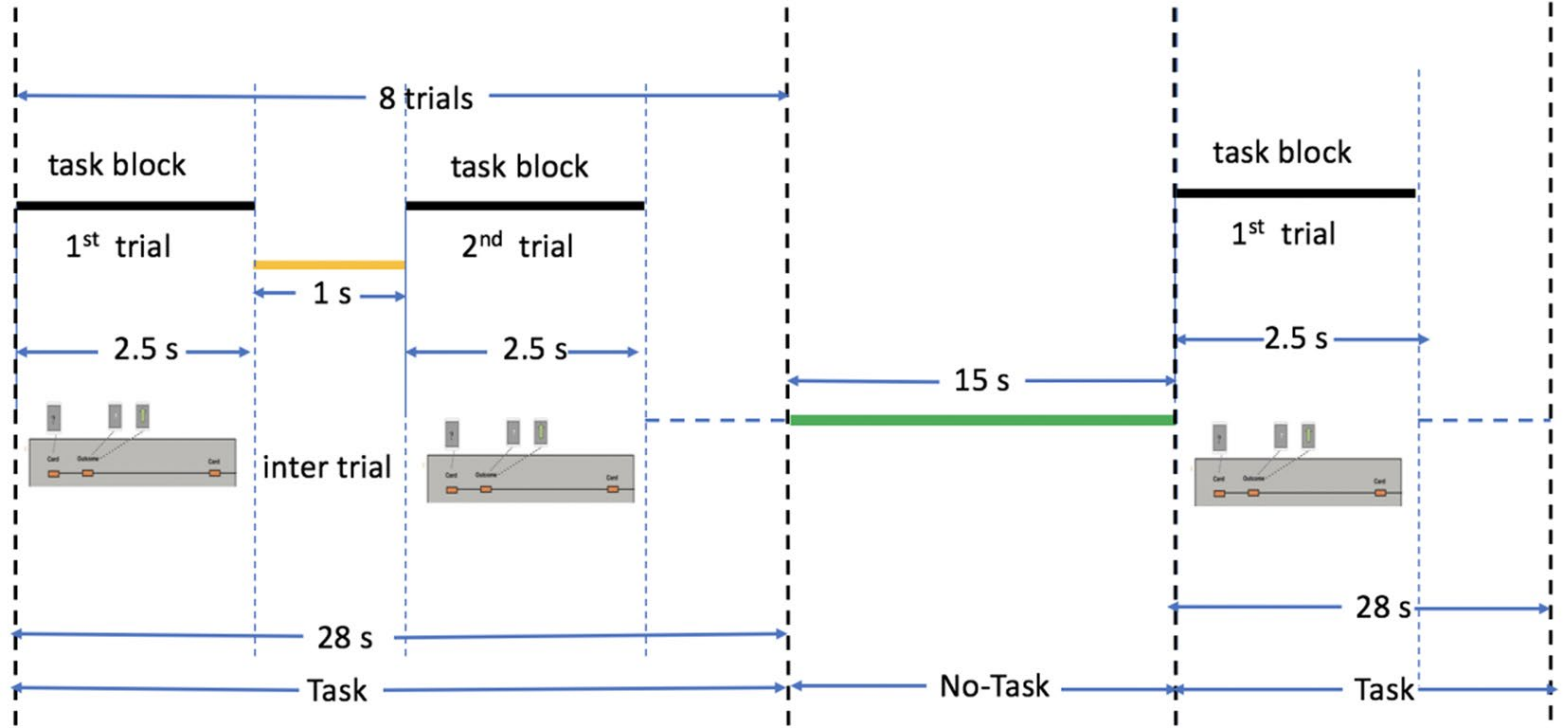
Gambling task pipeline. Each task block consists of 8 trials of gambling task paradigm following^78^ of duration 28 s. This follows by a no-task block of 15 s. There are 4 task blocks and 4 no-task blocks in total.

This task was adapted from the one developed by Delgado *et al*.^78^. Participants play a card guessing game where they are asked to guess the number on a mystery card (represented by a question mask “?”) in order to win or lose money. Participants are told that potential card numbers range from 1–9 and to indicate if they think the mystery card number is more or less than 5 by pressing one of two buttons on the response box. Feedback is the number on the card (generated by the program as a function of whether the trial was a reward, loss or neutral trial) and either: 1) a green up arrow with “$1” for reward trials, 2) a red down arrow next to -$0.50 for loss trials; or 3) the number 5 and a gray double headed arrow for neutral trials. The “?” is presented for up to 1500 ms (if the participant responds before 1500 ms, a fixation cross is displayed for the remaining time), followed by feedback for 1000 ms. There is a 1000 ms inter-task interval with a “+” presented on the screen. The task is presented in blocks of 8 trials that are either mostly reward (6 reward trials pseudo randomly interleaved with either 1 neutral and 1 loss trial, 2 neutral trials, or 2 loss trials) or mostly loss (6 loss trials pseudo-randomly interleaved with either 1 neutral and 1 reward trial, 2 neutral trials, or 2 reward trials). In each of the two runs, there are 2 mostly reward and 2 mostly loss blocks, interleaved with 4 fixation blocks (15 seconds each). The task format is shown in Fig. 7. The task is described based on *WU-Minn HCP* 500 *Subjects Data Release Manual* available from https://www.humanconnectome.org/.

### Prepossessing

**Figure 8:**
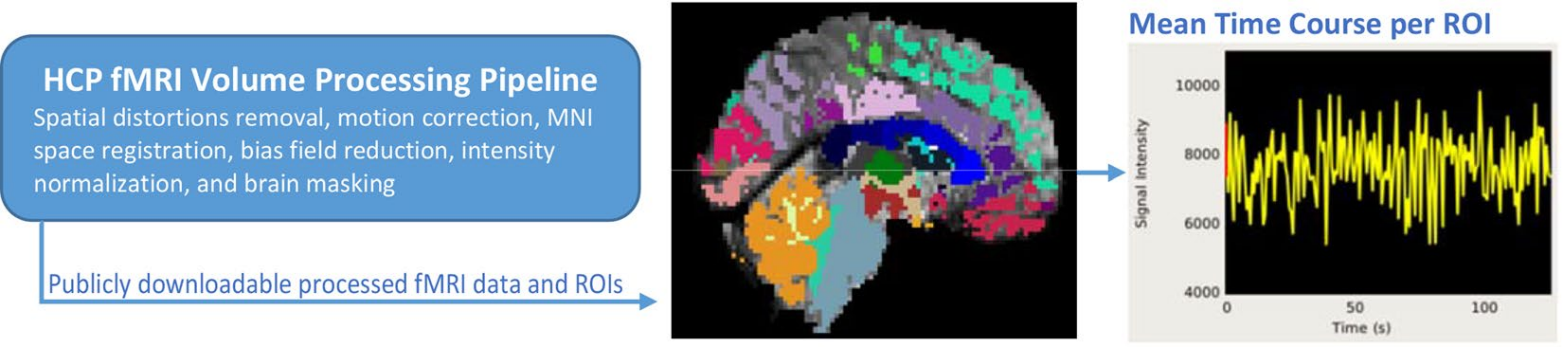
Preprocessing pipeline for extraction of fMRI time-series corresponding to anatomical regions.

The HCP task-fMRI data was first processed following the HCP “fMRIVolume” pipeline (v3.4)^79^, which includes gradient unwrapping, motion/distortion correction, registration to structural scan, nonlinear registration into MNI152 space, and intensity normalization as reported in^9^. Subsequently, spatial smoothing and activation maps generation using the generalized linear model implemented in FSL’s FILM (FMRIB’s Improved Linear Model with autocorrelation)^80^ were performed. Additional details about the HCP “fMRIVolume” pipeline can be found in Barch *et al*.^76^. Using Freesurfer cortical parcellation atlas^38^, 85 regions of interest were identified as shown in Table S5 in Supplementary Information. An illustration of this pipeline is shown in Fig. 8. Mean time-series value of voxels in every region for each subject were then extracted separately for task and no-task conditions. The task blocks (respectively no-task blocks) were concatenated for each subject and for each region corresponding to task (respectively no-task). Also, linear, square and cubic trends were removed from these time-series.

### Modeling the Brain Graph from fMRI

**Figure 9:**
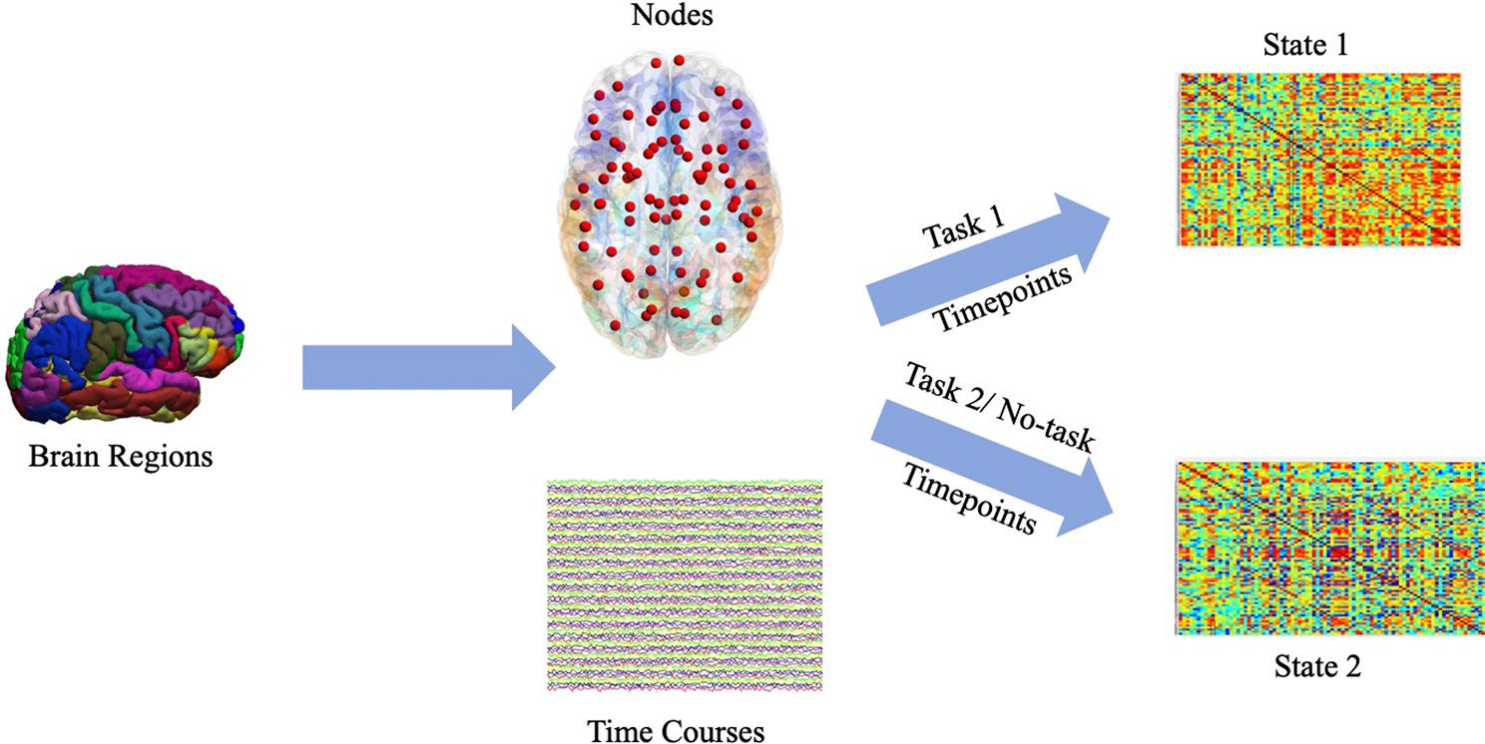
Pipeline for associating node and time-series to *states*.

After mean time-series are extracted from predefined anatomical regions^38^ from fMRI, a matrix of *R* × *T* (note that *R* = |*V*|) is generated. Here *R* is the number of regions and *T* is the number of time points. A node in the brain graph corresponds to a region of interest and is associated with one mean time-series. Absolute value of Pearson *correlation coefficient* between two mean time-series represents the edge weight associated with two nodes. This makes sure that we only have positively correlated edges. Absolute value of Pearson *correlation coefficient*s are computed separately for task *states* and no-task *states* as defined before. Specifically, the network connectivity for a task is constructed from fMRI time points when a subject is performing a task (*e*.*g*., emotion, gambling) during a t-fMRI experiment^12–15^. The network connectivity for a no-task is constructed from fMRI time points when a subject is *not* performing a task during a t-fMRI experiment^17^. Hence we get two adjacency matrices for each subject. The mapping process is shown in Fig. 9. Each adjacency matrix is made sparse by keeping top correlating edges. The edges had the same sparsity for all subjects. This was done by choosing $S=\frac{{\mathrm{log}}_{2}(R)}{{\mathrm{log}}_{2}(k)}=1.8$ where *k* is average degree in the graph^22^.

### Centrality Measures

Throughout our analysis, we assume that an un-directed brain network is given by *G* = (*V*, *E*) where *V* contains vertices or nodes, *E* contains weighted edges. Number of nodes is given by |*V*| which is equal to number of regions or neuronal units (R). Number of edges is given by |*E*|. In this section, first we define graph entropy based on the edge weights of graph.

#### Edge Weight of Graph

The edge weight *e*_*ij*_ between two nodes (*v*_*i*_, *v*_*j*_) is defined by the absolute value of Pearson *correlation coefficient* between their corresponding time-series. Thus the measure of edge weight *e*_*ij*_ is proportional to the magnitude of correlation between the two time-series (*v*_*i*_, *v*_*j*_) as defined by *ρ*(*i*, *j*) = *E*[*v*_*i*_*v*_*j*_] − *E*[*v*_*i*_]*E*[*v*_*j*_], where *E*[*X*] represents average value of random variable *X*. This implies that if *e*_*ij*_ is higher, the two nodes behave more similarly, i.e., their interaction is more. Hence, the probability of communication between *v*_*i*_, *v*_*j*_ is proportional to *e*_*ij*_. We used 4 types of centrality measures for comparison namely *degree*, *betweenness*, *eigenvector and leverage*^22^.

#### Degree Centrality

Degree^24^ of node *i* is determined by the number of neighbors connected to node *i*.

#### Eigenvector Centrality

Eigenvector centrality^25^ *e*_*i*_ is calculated by Equation 1.

1 $${e}_{i}=\frac{1}{\lambda }\sum _{j=1}^{R}\,{a}_{ij}{e}_{j}$$

Here *a*_*i*,*j*_ is (*i*, *j*)^*th*^ entry of adjacency matrix corresponding to the graph and *λ* is a constant.

#### Betweenness Centrality

Betweenness Centrality^25^ of node *i*, *b*_*i*_, is defined by the Equation 2.

2 $${b}_{i}=\frac{1}{(N-\mathrm{1)(}N-\mathrm{2)}}\sum _{x}\,\sum _{y}\,\frac{{g}_{xiy}}{{g}_{xy}}$$

Here *g*_*xy*_ is the number of shortest paths between any two nodes *x* and *y*. Also *g*_*xiy*_ is the number of paths among those passing through node *i*.

#### Leverage Centrality

Leverage centrality *l*_*i*_ is a measure of the relationship between the *degree* of a given node (*k*_*i*_) and the *degree* of each of its neighbors (*k*_*j*_), averaged over all neighbors (*N*_*i*_) as reported in^22^, and is defined in Equation 3.

3 $${l}_{i}=\frac{1}{{k}_{i}}\sum _{{N}_{i}}\,\frac{{k}_{i}-{k}_{j}}{{k}_{i}+{k}_{j}}$$

The following two metrics are used for statistical comparison with graph entropy metrics.

#### Generalized Linear Model

Generalized linear model^23^ is multiple regression of event blocks onto fMRI time-series. If there are two conditions, *e*.*g*., task and no-task, the regression coefficients are estimated for each condition on each time-series. Their differences describe the activation map for each region. The regression coefficients are computed based on ordinary least square technique^81^.

#### Structural Centrality

Structural centrality^82^ of a network is defined as

4 $$C(G)=\frac{4R(R-\mathrm{1)}}{{R}^{2}-4}\sum _{i\mathrm{=1}}^{R}\,{q}_{i,j}\sum _{j\mathrm{=1,}i\ne j}^{R}\,[{q}_{i,j}-\frac{1}{R(R-\mathrm{1)}}]$$

where *R* is the number of nodes. If *C*(*G*) is high, then the network is more central, i.e., they are influenced by a few leading nodes. A comparison of structural centrality and node entropy is shown in Subsection S.7 in Supplementary Information.

### Proposed Metric: Graph Entropy

#### Entropy of Graph

For graph *G* = (*V*, *E*), let two nodes be *v*_*i*_ and *v*_*j*_. The weight of the edge between two nodes *v*_*i*_, *v*_*j*_ is denoted by *e*_*ij*_. Here *e*_*ij*_ represents the absolute value of the correlation coefficient of the two time-series associated with time-series of regions v_i_ and v_j_ and specifies the interaction between two nodes (*v*_*i*_, *v*_*j*_). Let

5 $${q}_{i,j}=(\begin{array}{c}\begin{array}{ll}\frac{{e}_{ij}}{\sum ({e}_{ij}\in E)} & {\rm{when}}\,i\ne j,\\ 0 & {\rm{when}}\,i=j,\end{array}\end{array}$$

where *q*_*i*,*j*_ is probability of correlation between nodes (*v*_*i*_, *v*_*j*_). It is easy to see that ${\sum }_{i,j}\,{q}_{i,j}=1$. Note that *q*_*i*,*j*_’s can also be identified as entries in the normalized incidence matrix *Q* of graph *G* such that *Q*(*i*, *j*) = *q*_*i*,*j*_.

This definition allows us to define the *graph entropy* as

6 $$H(G)=-\sum _{\begin{array}{c}{e}_{ij}\in E\\ {q}_{i,j}\ne 0\end{array}}\,{q}_{i,j}{\mathrm{log}}_{2}({q}_{i,j}\mathrm{).}$$

*H*(*G*) can be seen as total amount of uncertainty in the whole network and its unit is bits. This entropy measure was introduced in^35^. Graph entropy has an inverese relationship with respect to *structural centrality*^82^.

Some mathematical properties of graph entropy as in Eq. 6 that are of interest are listed below.

- If some *q*_*i*,*j*_ = 1, then *H*(*G*) = 0. In that case, region *i* always communicates with region *j*. No other regions communicate with each other. Here *i*, *j* are leader nodes in the network.
- *H*(*G*) takes its maximum value when all *q*_*i*,*j*_’s are equally distributed. Here all regions participate equally in the communication process and the system is a homogeneous system. Hence, $H(G)\approx {\mathrm{log}}_{2}R(R-\mathrm{1)}$. In this scenario, no node is leader.
- The more uniform the distribution of the values of *q*_*i*,*j*_, for any given number of communication channels, the larger the value of *H*(*G*). That is, *H*(*G*) is larger for those cases where there is no communication dominance or ranking of the participants. Communication dominance reduces the graph entropy in *G*.

#### Entropy of Sub-Graphs

*Sub-graphs* can denote any portion or sub-structure of the main graph. For a *sub-graph G*_*s*_ = (*V*_*s*_, *E*_*s*_), the modified incidence matrix *Q*′ can be computed using Eq. 7.

7 $${q^{\prime} }_{k,m}=(\begin{array}{c}\begin{array}{ll}\frac{{e^{\prime} }_{km}}{\sum ({e^{\prime} }_{km}\in {E}_{s})} & {\rm{when}}\,k\ne m,\\ 0 & {\rm{when}}\,k=m.\end{array}\end{array}$$

The *sub-graph* entropy can be computed as follows,

8 $$H({G}_{s})=-\sum _{\begin{array}{c}k,m\in {V}_{s}\\ {q^{\prime} }_{k,m}\ne 0\end{array}}\,{q^{\prime} }_{k,m}{\mathrm{log}}_{2}({q^{\prime} }_{k,m})$$

This measure is comparable among different *sub-graph*s of the same graph as the edges are normalized before computation of entropy.

#### Node Entropy

Let *v*_*i*_ be a node in graph *G*. Also, let *sub-graph* ${G}_{{v}_{i}}$ contain the node *v*_*i*_ and its immediate 1-hop neighbors. Specifically, ${G}_{{v}_{i}}$ consists of node *v*_*i*_ and the neighboring nodes that can be reached from *v*_*i*_ through its edges by hopping only once. Now let the set of nodes in ${G}_{{v}_{i}}$ be ${V}_{{v}_{i}}$ and the edges be the 1-hop edges from *v*_*i*_ denoted by ${E}_{{v}_{i}}$. Then the entries in modified incidence matrix can be calculated as

9 $${q^{\prime} }_{k,m}=(\begin{array}{c}\begin{array}{ll}\frac{{e^{\prime} }_{k,m}}{\sum ({e^{\prime} }_{k,m}\in {E}_{{v}_{i}})} & {\rm{when}}\,{k}\ne m,\\ 0 & {\rm{when}}\,{k}=m,\end{array}\end{array}$$

where *q*′_*k*,*m*_ is the normalized correlation coefficient between nodes (*v*_*k*_, *v*_*m*_) within that *sub-graph*. We define node entropy as given by,

10 $$H({G}_{{v}_{i}})=-\sum _{\begin{array}{c}k,m\in {V}_{{v}_{i}}\\ {q^{\prime} }_{k,m}\ne 0\end{array}}\,{q^{\prime} }_{k,m}{\mathrm{log}}_{2}({q^{\prime} }_{k,m})$$

#### Edge Entropy

Let *e*_*ij*_ denote the edge between vertices (*v*_*i*_, *v*_*j*_). The *sub-graph* corresponding to this edge is defined by combining 1-hop *sub-graph*s of nodes *v*_*i*_ and *v*_*j*_. Assuming, ${G}_{{v}_{i}}=({V}_{{v}_{i}},{E}_{{v}_{i}})$ and ${G}_{{v}_{j}}=({V}_{{v}_{j}},{E}_{{v}_{j}})$, the new *sub-graph* for *e*_*ij*_ is ${G}_{{e}_{ij}}=({V}_{{e}_{ij}},{E}_{{e}_{ij}})$ where ${V}_{{e}_{ij}}=({V}_{{v}_{i}}\cup {V}_{{v}_{j}})$ and ${E}_{{e}_{ij}}=({E}_{{v}_{i}}\cup {E}_{{v}_{j}})$. Then the entries in modified incidence matrix can be calculated as

11 $${q^{\prime} }_{k,m}=(\begin{array}{c}\begin{array}{ll}\frac{{e^{\prime} }_{k,m}}{\sum ({e^{\prime} }_{k,m}\in {E}_{{e}_{ij}})} & {\rm{when}}\,k\ne m,\\ 0 & {\rm{when}}\,k=m,\end{array}\end{array}$$

where *q*′_*k*,*m*_ represents the normalized correlation coefficient between nodes (*v*_*k*_, *v*_*m*_) within that *sub-graph*. We define edge entropy as given by,

12 $$H({G}_{{e}_{ij}})=-\sum _{\begin{array}{c}k,m\in {V}_{{e}_{ij}}\\ {q^{\prime} }_{k,m}\ne 0\end{array}}\,{q^{\prime} }_{k,m}{\mathrm{log}}_{2}({q^{\prime} }_{k,m})$$

### Statistical Analysis

The node entropy and edge entropy values are compared across different *states* for all subjects. Based on their differences in entropy, they are ranked in descending order. We also calculate their corresponding *p-values* using a permutation t-test. The regions with significant change in entropies (*p* ≤ 0.05) are illustrated in a table. The edges with significant change in entropies (*p* ≤ 0.05), are plotted as sub-network in a brain template. To understand if the chosen rankings were stable enough, a leave-one-out subject scheme was implemented to select top regions and edges. In particular, in each iteration one subject is left out and the regions, edges are ranked based on the other 474 subjects. The occurrence of the most important regions and edges were plotted in a histogram^41–43^. To quantify the significance of classification performance, permutation tests are performed. This involves computing a trivial baseline–the accuracy produced by permuting the labels and then determining if the learned model performed significantly better than that. Here, we perform 1000 iterations for each of the datset, then we train a model on the training data and test it on the remaining instances. The classification performance of the proposed model is also compared with baseline methods using binomial tests. This involves using the baseline accuracies as parameter of a binomial distribution and calculating the probability of achieving the accuracy achieved by the proposed models.

In addition, graph entropy values for regions were correlated with other four centrality measures. We create a scatter plot containing regional entropy values *vs*. each of *degree*, *betweenness*, *eigenvector*, *leverage*. The correlation values between node entropy and other centralities for each subject are calculated. The total graph entropy measures were used to differentiate between task *vs*. no-task condition. We use t-test and effect size to differentiate these two *states* at a group-level. Furthermore, node and edge entropy values are compared using our algorithm and top-25 values are used to classify task *vs*. no-task *states* in fMRI scan in each case (region, edge).

### Software

MATLAB is used for running experiments and generating the results. Custom MATLAB code is created for extracting graph entropy measures. We used the brain connectivity toolbox (BCT)^3^ to calculate the centrality metrics. SVM classifiers are designed using LIBSVM toolbox^83^.

## Conclusion

The main contribution of the study is to demonstrate that well defined brain *states* can be predicted using *sub-graph* entropy from t-fMRI data. We showed that there are *important* nodes and edges in *functional connectivity* that are sufficiently distinguishing between two different brain *states*. This paper has introduced the notion of *sub-graph* entropy in general and node and edge entropies in particular to rank regions and edges in brain graphs in a quantitative manner. Results obtained by the proposed method have been compared with that from the generalized linear model (GLM), *degree* centrality, *eigenvector* centrality, *betweenness* centrality and *leverage* centrality and network based statistics (NBS). In this paper, node and edge entropies have been defined based on 1-hop neighbors. Whether node and edge entropies defined using 2-hop neighbors provide more accurate prediction of brain network state needs further research. Future work will be directed towards applications of the technique in identifying dynamic states from fMRI tasks as well as from other temporally rich signals such as electroencephalogram (EEG)^84,85^ and magnetoencephalogram (MEG)^86,87^. While node and edge entropies have been used in this paper, identifying *sub-graphs* corresponding to certain tasks requires further research. Investigating applications of the technique to understand differences in brain networks of populations with various diseases and healthy control is also of interest. In many disease prediction applications, filtered versions of time-series have been found to be more discriminative of the disease state^42–44,88^. Thus, sub-graph entropy features should be extracted from filtered fMRI and then used for classification; this topic needs to be investigated further.

## Supplementary information

Supplementary Information

## Data Availability

The datasets analyzed for this study are available to the public from the Human Connectome Project (Open Access Data) ConnectomeDB database.
