# Supplementary material for: Ranking Regions, Edges and Classifying Tasks in Functional Brain Graphs by Sub-Graph Entropy
Source: Sci Rep. 2019 May 20;9:7628. doi: 10.1038/s41598-019-44103-8 (PMC6527859; doi:10.1038/s41598-019-44103-8)
Supplement: Supplementary file 1 — Supplementary Information [file 41598_2019_44103_MOESM1_ESM.pdf]

Ranking Regions, Edges and Classifying Tasks in Functional Brain Graphs by Sub-Graph Entropy

Bhaskar Sen[1], **Shu Hsien Chu**[1] **and Keshab K. Parhi**[1,*]

[1]Electrical and Computer Engineering, University of Minnesota, Minneapolis
[*]parhi@umn.edu

Supplementary Information

The Supplementary Information contains twelve Subsections. Subsection S.1 provides pipeline for the ranking process. Subsection S.2 illustrates the important regions and edges for different task conditions. Subsection S.3 illustrates the ROC curves for classification performance. Subsection S.4 lists edges based on differential edge entropy and lists ranked regions by state-of-the-art centrality measures. Subsection S.5 shows the histograms of occurrences for regions and edges. Subsection S.6 shows distribution of accuracy values during a permutation test. Subsection S.7 compares graph entropy with structural centrality. Subsection S.8 contains images containing comparison of graph entropy and sub-graph (intersection and union of top regions and edges) entropies between two groups. Subsection S.9 describes the details about the atlas used in this paper. Subsection S.10 shows that maximizing *sub-graph* entropy is equivalent to maximizing mutual information. Subsection S.11 contains a theoretical justification for using sample average entropy as proxy for group entropy. Subsection S.12 provides a justification of using absolute correlation as edge weights.

S.1: Pipeline for Ranking of Regions and Edges

This subsection illustrates the pipeline for ranking procedure of nodes and edges.

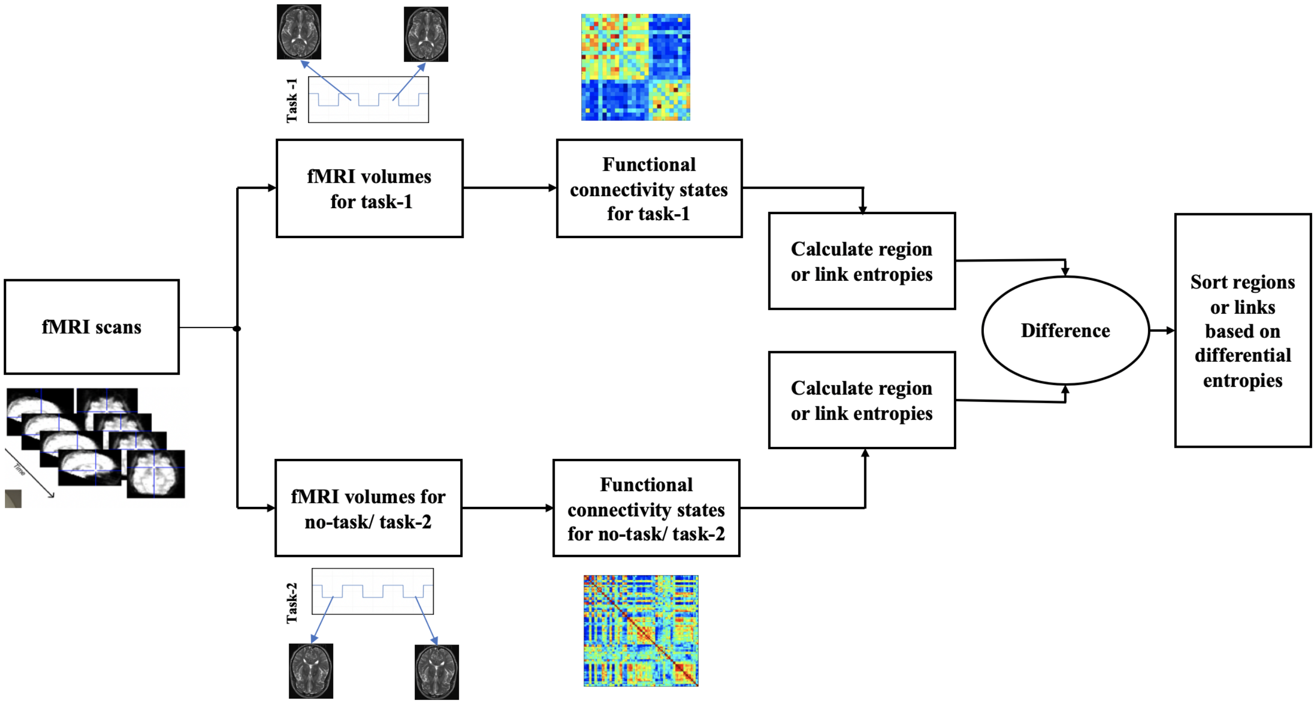

Figure S1. Pipeline for ranking of regions and edges. After parcellating fMRI scans into regions, *sub-graph* entropies (region and edge entropies) are calculated for each subject's functional network. These differential entropies are then used for ranking the regions and edges.

S.2: Important Regions and Edges

This subsection demonstrates the important nodes and edges for different *states*.

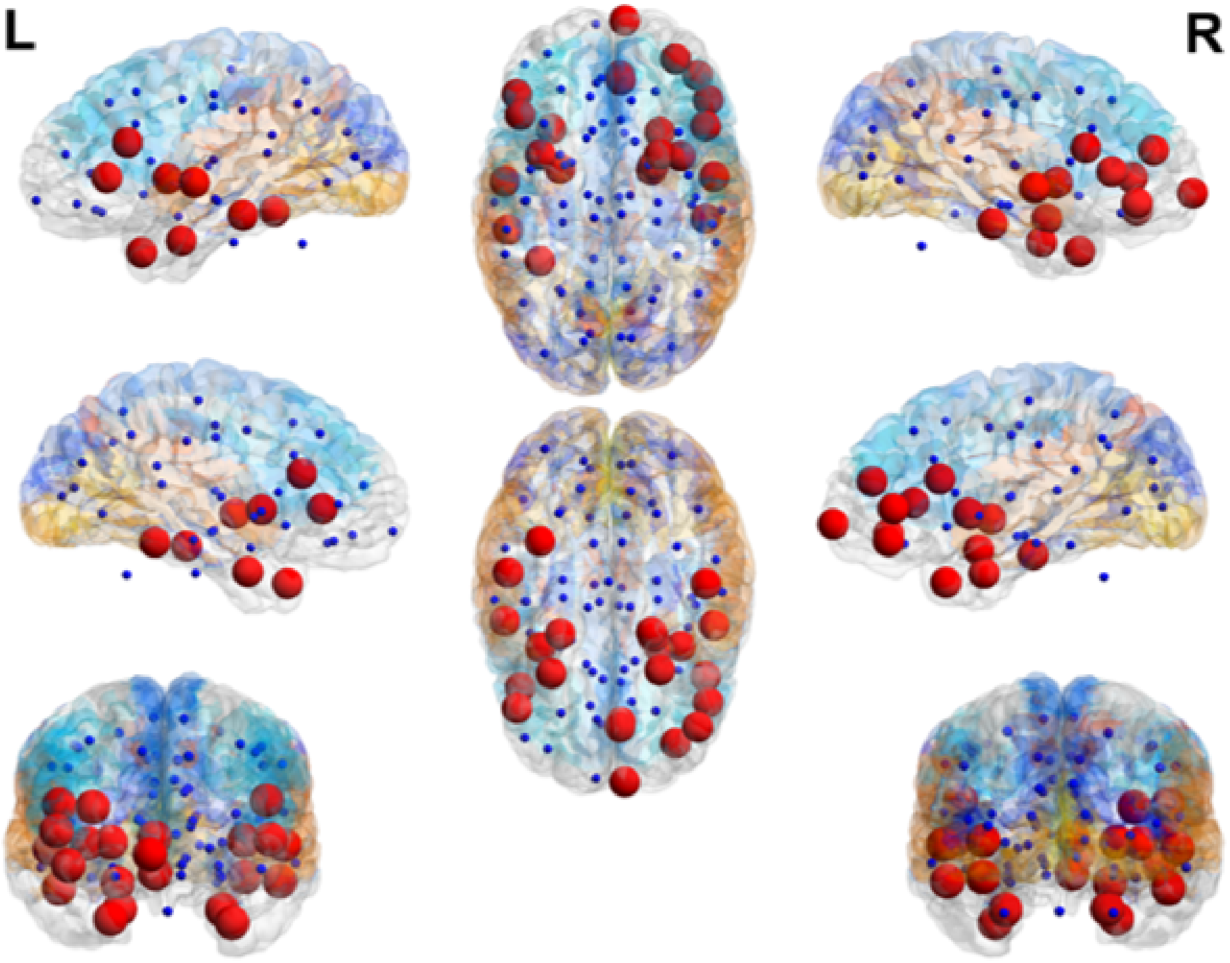

Figure S2. Visualization of important regions within brain. These regions carry the highest entropies within the functional network related to a *task* representing emotion task. The regional importance is shown by the size of spheres. Top-25 regions are shown in *red*. All regions are at least 2-standard deviations away from mean entropy.

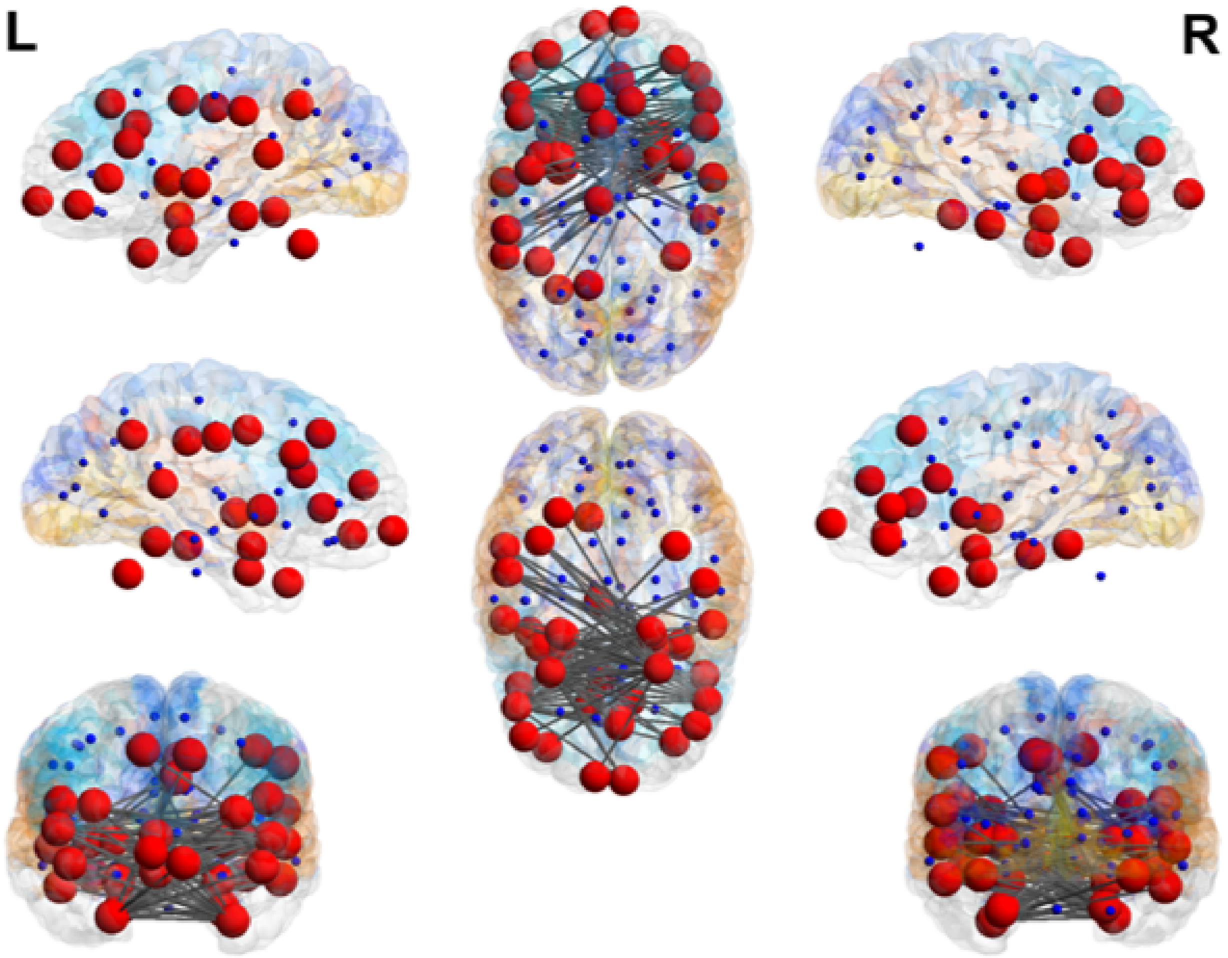

Figure S3. Visualization of important edges within brain for emotion task. These edges carry the highest entropies within the functional network related to a *task*. The regional importance is shown by the size of spheres. Top-25 regions that are at least 2-standard deviations away from mean entropy are shown in *red*.

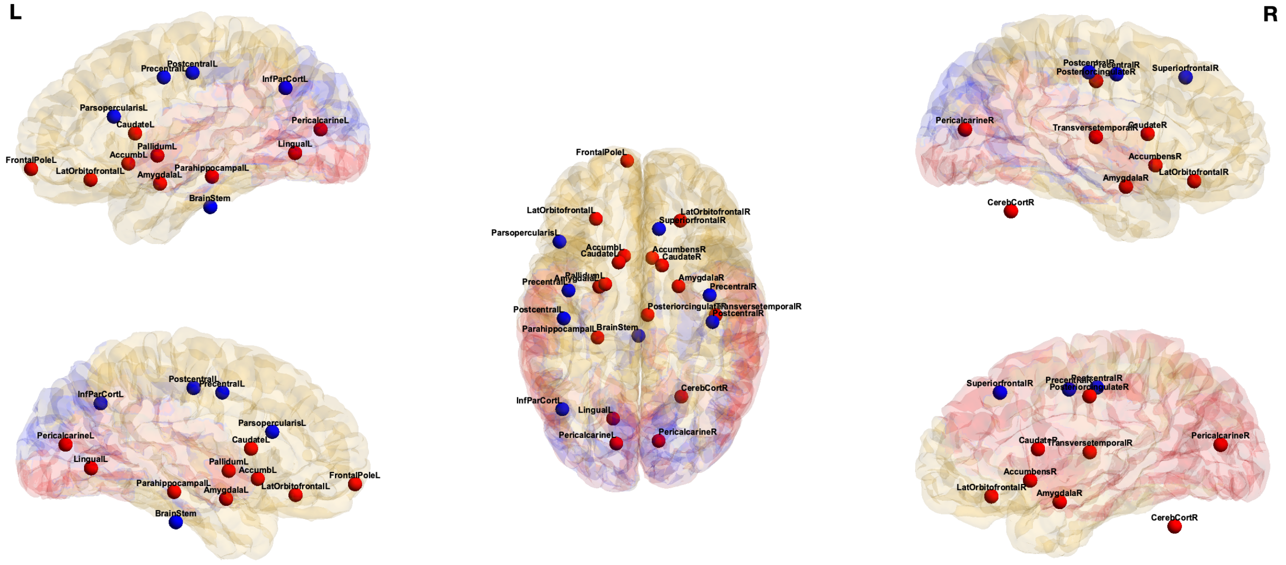

Figure S4. Visualization of *important* regions that have highest differential entropy between two *states* for gambling task *vs.* no-task. *Red:* regions that have higher node entropy during gambling task, *blue:* regions that have higher node entropy during no-task.

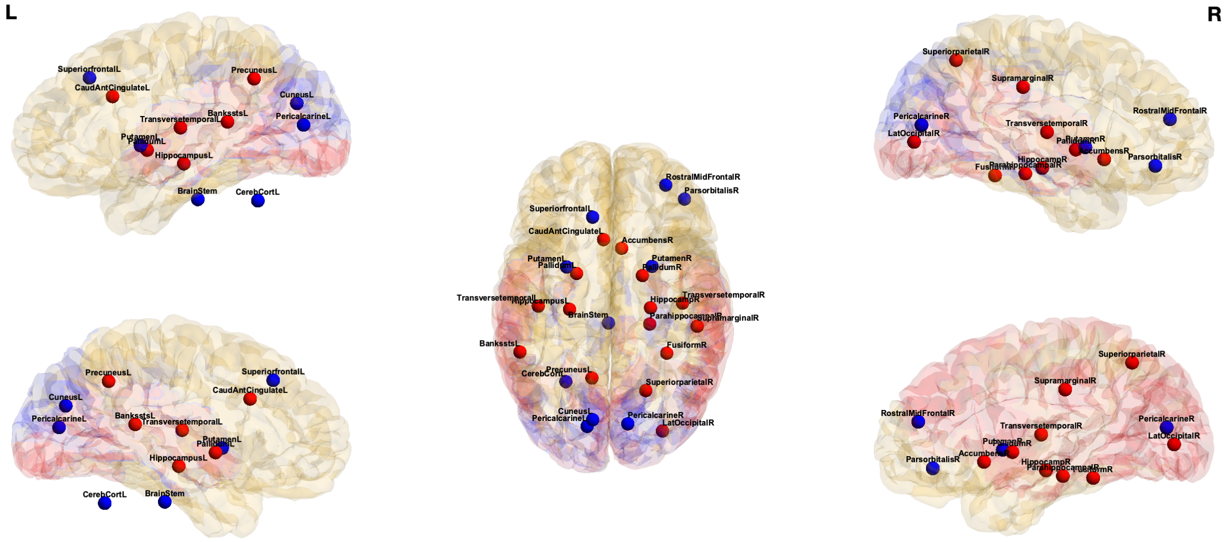

Figure S5. Visualization of *important* regions that have highest differential entropy between two *states* for emotion *vs.* gambling task. *Red:* regions that have higher node entropy during emotion task, *blue:* regions that have higher node entropy during gambling task.

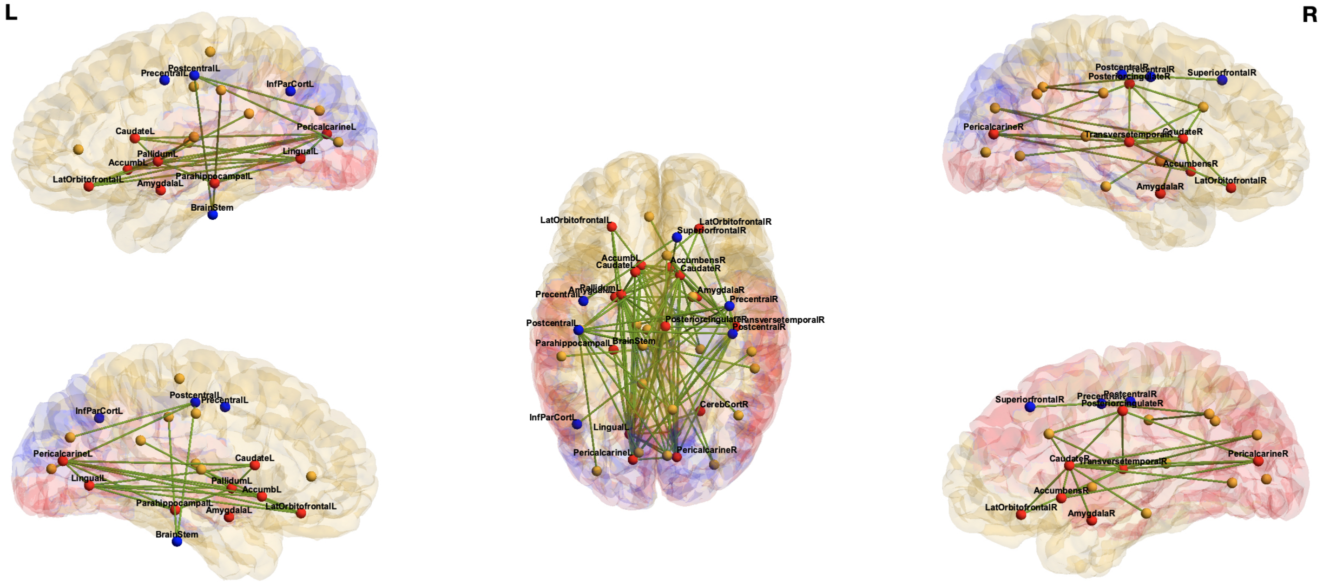

Figure S6. Visualization of *important* edges that have highest differential entropy between two *states* for gambling task *vs.* no-task. *Red:* regions that have higher node entropy during gambling task, *blue:* regions that have higher node entropy during no-task, *yellow:* regions that are not significant based on only node entropy.

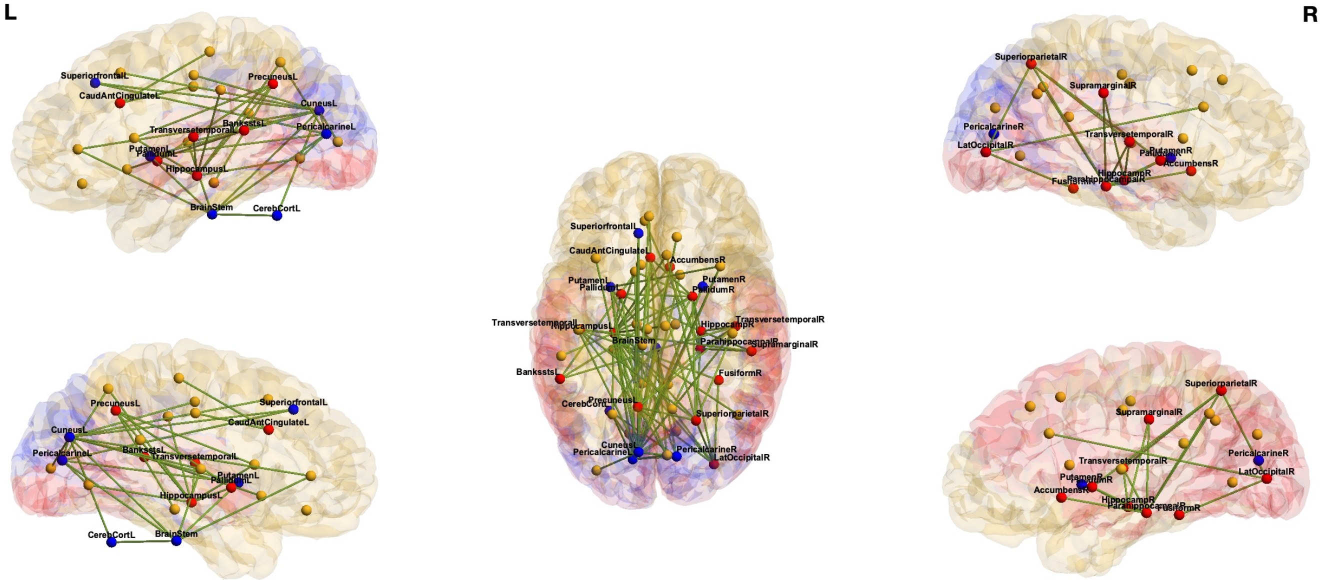

Figure S7. Visualization of *important* edges that have highest differential entropy between two *states* for emotion *vs.* gambling task. *Red:* regions that have higher node entropy during emotion task, *blue:* regions that have higher node entropy during gambling, *yellow:* regions that are not significant based on only node entropy.

S.3: ROC curve for classification

This subsection compares the Receiver Operating Characteristic (ROC) curves for different classification tasks.

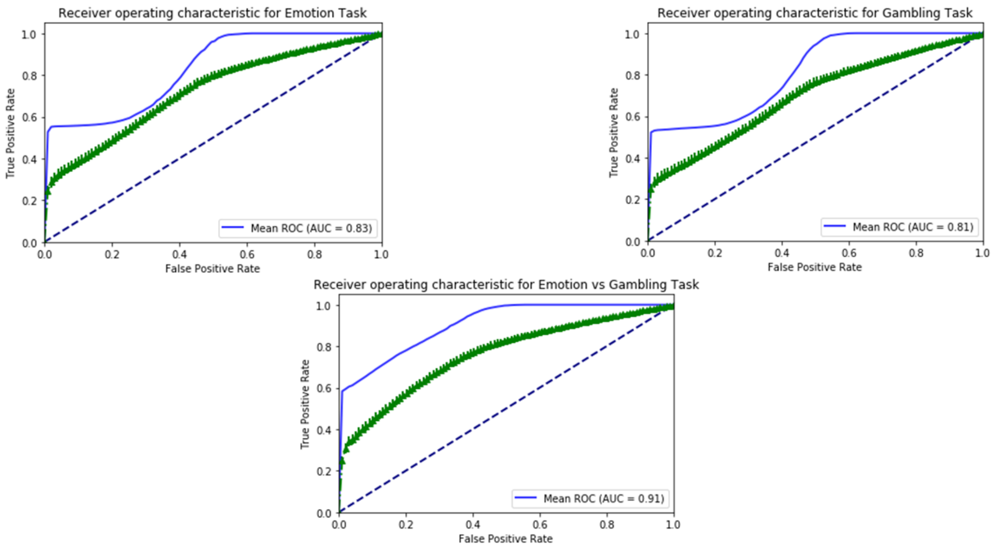

Figure S8. ROC curve for region based centrality. *blue:* ROC curve using sub - graph entropy, *green:* mean ROC curve using 4-centrality measures: *degree*, *eigenvector*, *betweenness*, *leverage*, *blue broken:* random guessing using ROC baseline.

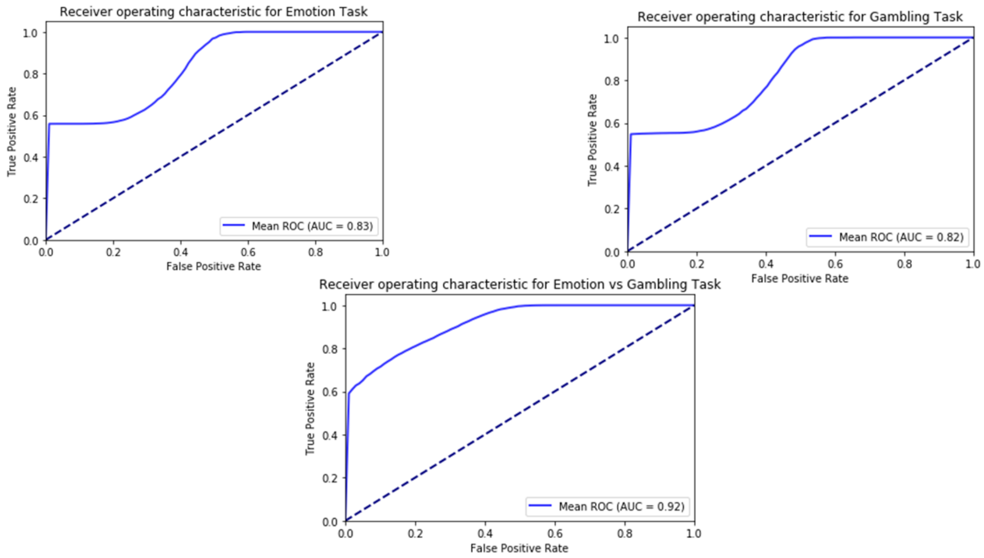

Figure S9. ROC curve for edge based centrality: *blue:* ROC curve using *sub-graph* entropy, *blue broken:* ROC baseline using random guessing.

S.4: Edge Ranking Based on Differential Entropy

This subsection lists the edges ranked according to their differential entropy.

Table S1. Edge ranking

Emotion *vs.* No-task			Gambling *vs.* No-task		
Edges		Entropy	Edges		Entropy
ctx_lh_pericalcarine	ctx_rh_pericalcarine	0.1917	Left_Pallidum	Right_Caudate	0.1834
ctx_lh_pericalcarine	ctx_lh_superiorparietal	0.1884	ctx_lh_lingual	ctx_lh_pericalcarine	0.1816
ctx_lh_superiorparietal	ctx_rh_superiorparietal	0.1838	ctx_rh_lateralorbitofrontal	ctx_rh_transversetemporal	0.1758
ctx_lh_superiorparietal	ctx_rh_pericalcarine	0.1843	Left_Accumbens_area	ctx_rh_transversetemporal	0.1647
ctx_lh_pericalcarine	ctx_rh_superiorparietal	0.1778	Left_Caudate	ctx_lh_pericalcarine	0.1641
Right_Pallidum	ctx_lh_isthmuscingulate	0.1997	Left_Accumbens_area	ctx_rh_parahippocampal	0.1613
ctx_rh_pericalcarine	ctx_rh_superiorparietal	0.1495	Left_Pallidum	ctx_lh_pericalcarine	0.1605
Right_Thalamus_Proper	ctx_lh_superiorparietal	0.1431	Left_Caudate	Left_Pallidum	0.1562
ctx_lh_pericalcarine	ctx_rh_lingual	0.1406	ctx_lh_pericalcarine	ctx_rh_transversetemporal	0.15422
ctx_lh_superiorparietal	ctx_rh_lingual	0.1404	ctx_lh_postcentral	ctx_rh_precuneus	0.1515
ctx_lh_pericalcarine	ctx_rh_cuneus	0.1384	Left_Pallidum	ctx_lh_lingual	0.1487
Left_Accumbens_area	Right_Pallidum	0.1382	Left_Caudate	Right_Caudate	0.1484
Right_Caudate	Right_Pallidum	0.1335	Right_Caudate	ctx_rh_parahippocampal	0.1468
ctx_lh_rostralanteriorcingulate	ctx_lh_superiorparietal	0.1312	Left_Accumbens_area	ctx_lh_pericalcarine	0.1463
ctx_lh_parahippocampal	ctx_rh_transversetemporal	0.1303	Left_Accumbens_area	Right_Accumbens_area	0.1443
ctx_lh_isthmuscingulate	ctx_rh_transversetemporal	0.1293	Left_Accumbens_area	Right_Caudate	0.1436
Right_Caudate	ctx_rh_transversetemporal	0.1285	Left_Pallidum	Left_Accumbens_area	0.1424
Right_Caudate	ctx_rh_parahippocampal	0.1273	Left_Pallidum	ctx_rh_cuneus	0.1398
Left_Hippocampus	Right_Pallidum	0.1268	Left_Pallidum	ctx_rh_transversetemporal	0.1392
Left_Hippocampus	ctx_rh_transversetemporal	0.1247	Right_Pallidum	ctx_lh_lingual	0.1391
ctx_rh_lingual	ctx_rh_pericalcarine	0.1240	Left_Pallidum	ctx_lh_parahippocampal	0.1390
ctx_rh_caudalanteriorcingulate	ctx_rh_transversetemporal	0.1228	ctx_lh_lateraloccipital	ctx_lh_postcentral	0.1388
ctx_lh_pericalcarine	ctx_rh_precuneus	0.1209	Right_Pallidum	ctx_lh_pericalcarine	0.1382
ctx_rh_lingual	ctx_rh_superiorparietal	0.1207	Left_Caudate	ctx_lh_lingual	0.1366
Left_Hippocampus	Left_Accumbens_area	0.1206	Right_Caudate	ctx_lh_pericalcarine	0.1357

Table S2. Edge rank emotion *vs.* gambling

Emotion *vs.* Gambling		
Edges		Entropy
Brain_Stem	ctx_lh_pericalcarine	0.7195
ctx_lh_cuneus	ctx_lh_pericalcarine	0.6580
Left_Hippocampus	Right_Pallidum	0.5991
Brain_Stem	ctx_lh_cuneus	0.5920
Left_Hippocampus	ctx_rh_caudalanteriorcingulate	0.5830
Left_Pallidum	Right_Pallidum	0.5671
Left_Caudate	Left_Hippocampus	0.5614
ctx_lh_pericalcarine	ctx_rh_pericalcarine	0.5489
Left_Hippocampus	ctx_lh_precuneus	0.5439
Brain_Stem	ctx_rh_lingual	0.5422
Left_Putamen	Brain_Stem	0.5317
Brain_Stem	ctx_rh_pericalcarine	0.5283
ctx_lh_cuneus	ctx_rh_pericalcarine	0.5147
ctx_lh_pericalcarine	ctx_lh_superiorfrontal	0.5099
ctx_lh_cuneus	ctx_lh_lateraloccipital	0.5054
Left_Hippocampus	Right_Accumbens_area	0.5026
Right_Hippocampus	ctx_rh_transversetemporal	0.4910
Right_Pallidum	ctx_rh_parahippocampal	0.4900
Left_Putamen	ctx_rh_pericalcarine	0.4800
Left_Hippocampus	ctx_lh_transversetemporal	0.4758
ctx_lh_cuneus	ctx_lh_lingual	0.4605
Left_Hippocampus	ctx_rh_lateraloccipital	0.4599
Left_Hippocampus	Right_Caudate	0.4597
ctx_lh_cuneus	ctx_lh_postcentral	0.4578
ctx_lh_precuneus	ctx_lh_transversetemporal	0.4561

Table S3. Significant regions extracted by other centrality measures between emotion *vs*. no-task.

Degree	Betweenness	Eigenvector	Leverage
PericalcarineL	PericalcarineL	AmygdalaR	AmygdalaR
SuperiorparietalL	PostcentralL	ParsopercularisL	PrecuneusL
FusiformR	PrecentralL	ParstriangularisL	FusiformR
PericalcarineR	PrecuneusL	PericalcarineL	HippocampusL
SuperiorparietalR	CuneusR	RostralMidFontalL	
RostralAntCingulateR	PericalcarineR	EntorhinalR	
	PrecuneusR	FusiformR	
		RostralAntCingulateR	
		RostralMidFrontalR	

Table S4. Significant regions extracted by other centrality measures between gambling *vs*. no-task.

Degree	Betweenness	Eigenvector	Leverage
PallidumL	HippocampusL	BrainStem	AmygdalaR
AccumbL	CerebCortR	AccumbL	ParsopercularisL
CaudateR	CaudMidFrontalL	CaudateR	AccumbL
AmygdalaR	InfTempCortL	LingualL	ParsorbitalisR
AccumbensR	LatOrbitofrontalL	ParacentralL	HippocampusL
MidTemporalL	MedOrbitofrontalL	ParstriangularisL	
ParsopercularisL		InsulaL	
PostcentralL		FusiformR	
PrecentralR		InfTempCortR	
SuperiorfrontalR		ParsorbitalisR	

S.5. Histogram

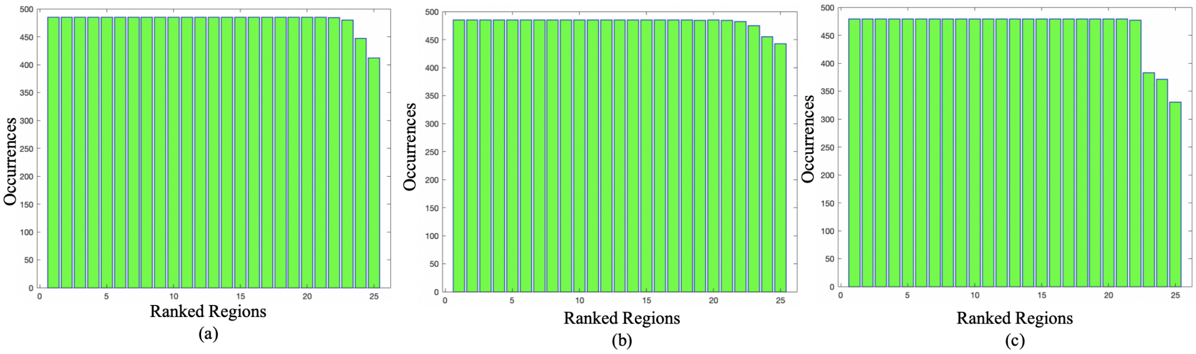

Figure S10. Histograms of occurrence for top-25 regions during leave-one-out ranking procedure. Algorithm 3 was run 475 times, each time leaving one subject out. Occurrence of top-25 regions among top-25 regions are plotted. The number represents ranked regions in Table 2. (a) Emotion *vs*. no-task, (b) gambling *vs*. no-task, (c) emotion *vs*. gambling.

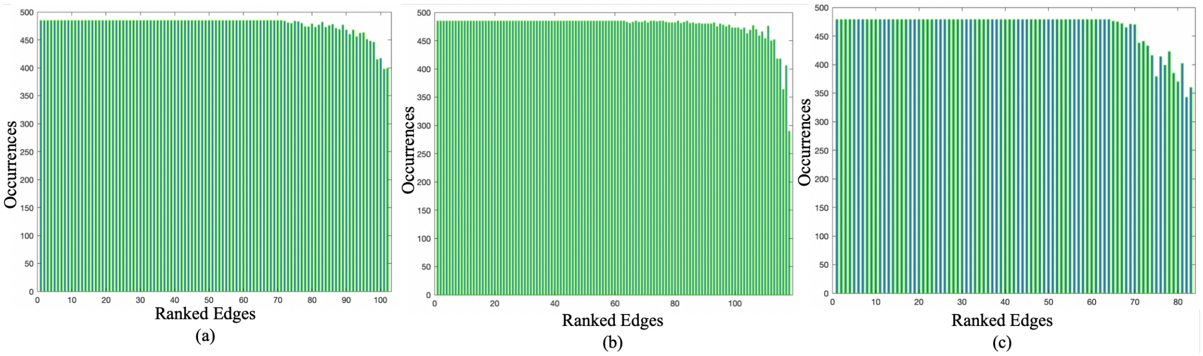

Figure S11. Histograms of occurrence for significant edges during leave-one-out ranking procedure. Algorithm 3 was run 475 times, each time leaving one subject out. Occurrence of significant edges are plotted. Edge numbers represent ranked edges in Table S1 and Table S2 in this Supplementary Information. (a) Emotion *vs*. no-task, (b) gambling *vs*. no-task, (c) emotion *vs*. gambling.

S.6. Distribution of Accuracy Values

This subsection contains the histograms for performing permutation test on the three classification tasks.

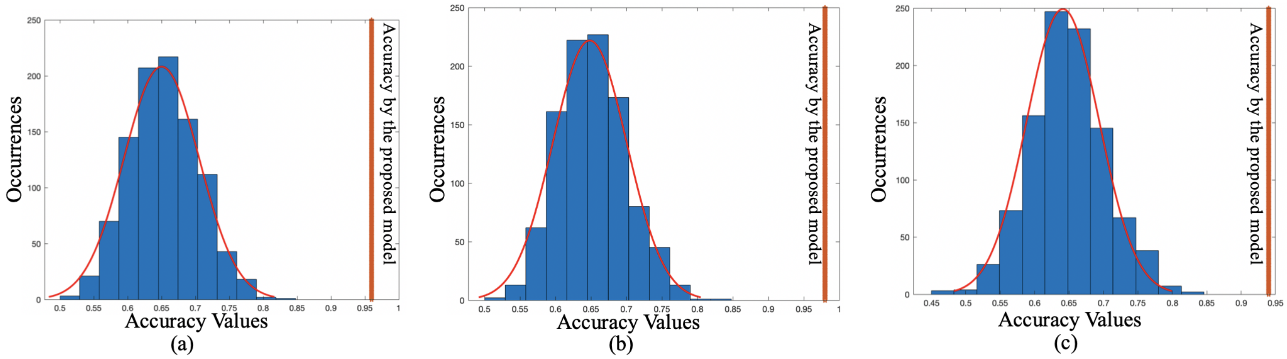

Figure S12. Permutation tests by randomly assigning labels. Results from 1000 iterations are shown in histogram. Results from proposed model are shown in *red* bar. (a) Emotion *vs*. no-task, (b) gambling *vs*. no-task, (c) emotion *vs*. gambling.

S.7. Structural Centrality and Graph Entropy

Graph entropy is related to *structural centrality* of a graph. In order to investigate the relationship between the two metrics, we simulate 100,000 random graphs consisting of 85 nodes. The edge values are created based on a uniform distribution between 0-1. For each graph, we calculate both *structural centrality* and graph entropy; these are plotted in Fig. S13. We also overlay the ranges for graph entropy for real brain graphs. These two quantities have an inverse relationship. As structural centrality of a graph increases, its entropy reduces. The opposite is true when entropy increases. A graph is more central and consists of one leader when entropy is less. On the other hand, the impact of nodes is more uniform when entropy of graph increases. Real fMRI datasets from Human Connectome Project consist of entropy values in the middle range.

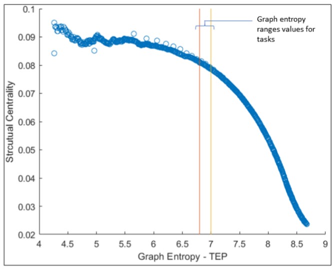

Figure S13. Structural centrality *vs.* graph entropy. Here 100 thousands random graphs consisting of 85 nodes were created of varying centrality. Each node graph entropy and structural centrality were calculated and plotted. The boundary for highest and lowest value for entropies of human brain network is shown in yellow lines.

S.8. Comparison of Graph Entropy and Sub-graph Entropy between Two Groups

This subsection contains images containing comparison of graph entropy and sub-graph (intersection and union of top regions and edges) entropies between two groups.

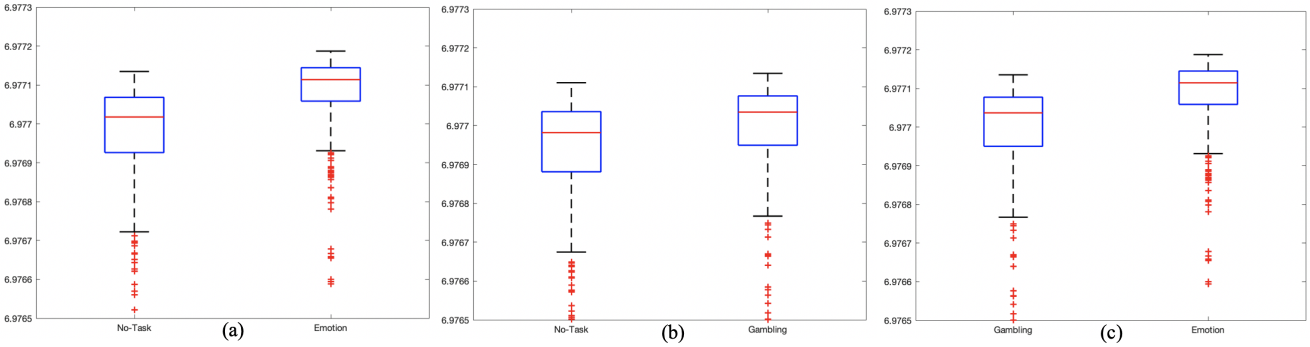

Figure S14. Comparison of group-level mean values for graph entropy for whole graph between two *states*. a) Emotion *vs.* no-task, b) gambling *vs.* no-task, c) emotion *vs.* gambling. The graph entropies are calculated for each subject and *state*.

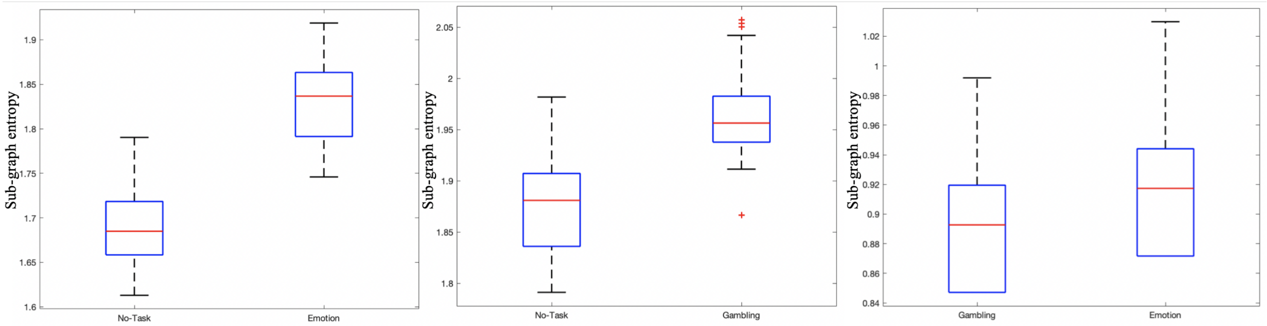

Figure S15. *Sub-graph - 1*: Comparison of group-level mean values for *sub-graph* entropy for sub-network containing the *intersection* of top-25 regions and significant edges between two *states*. a) Emotion *vs.* no-task (54 edges), b) gambling *vs.* no-task (64 edges), c) emotion *vs.* gambling (17 edges). The graph entropies are calculated for each subject and *state*.

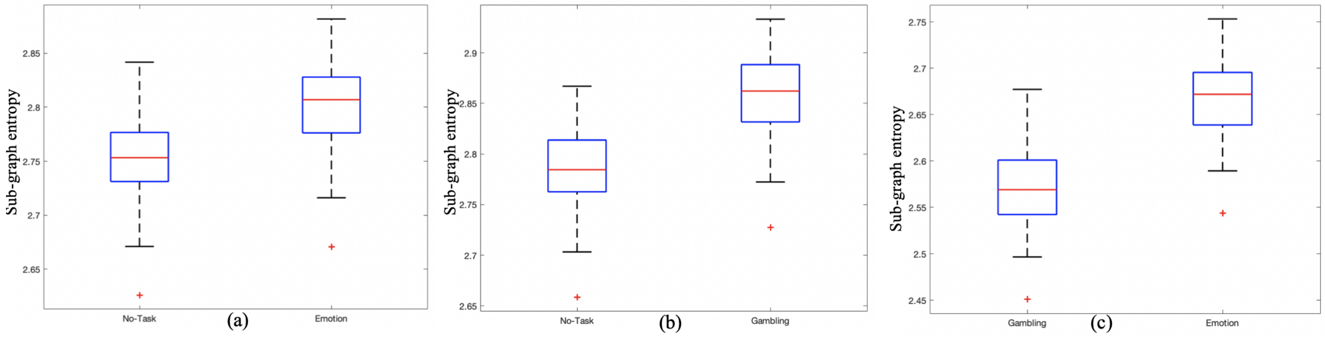

Figure S16. *Sub-graph - 2*: Comparison of group-level mean values for *sub-graph* entropy for sub-network containing *union* of top-25 regions and significant edges between two *states*. a) Emotion *vs*. no-task (102 edges), b) gambling *vs*. no-task (118 edges), c) emotion *vs*. gambling (83 edges). The graph entropies are calculated for each subject and *state*.

S.9: Atlas Details

This section provides the details of Freesurfer atlas regions and their corresponding coordinates in MNI152 space.

Table S5. Freesurfer Atlas Coordinates and Regions

X	Y	Z	Region
-22.43799052	-40.01410235	-49.10043329	Left_Cerebellum_Cortex
-9.365745637	2.239886304	-14.37367795	Left_Thalamus_Proper
-10.07260101	27.72348485	-12.26073232	Left_Caudate
-21.98422754	20.16952115	-20.84432891	Left_Putamen
-16.83028771	16.79043546	-23.15999222	Left_Pallidum
-0.075123916	-9.236922464	-48.42339352	Brain_Stem
-20.50547599	-2.062973884	-30.15532856	Left_Hippocampus
-19.81226823	15.48516687	-36.92135352	Left_Amygdala
-7.276182432	31.04307432	-27.10304054	Left_Accumbens_area
21.48534428	-39.86756307	-49.02782405	Right_Cerebellum_Cortex
9.483949985	2.061275227	-13.59662913	Right_Thalamus_Proper
11.78609914	26.20757004	-11.62715517	Right_Caudate
22.79048673	20.47876106	-20.71349558	Right_Putamen
17.75835396	15.67295792	-21.86726485	Right_Pallidum
22.00855593	-1.230696995	-31.1206177	Right_Hippocampus
20.08349546	15.711738	-37.17331388	Right_Amygdala
6.856174699	29.9811747	-26.78840361	Right_Accumbens_area
-46.56135363	-24.43048423	-8.671588408	ctx_lh_bankssts
-2.641475645	34.52602674	4.327244508	ctx_lh_caudalanteriorcingulate
-29.18799213	34.20815342	18.68062611	ctx_lh_caudalmiddlefrontal
-8.394193234	-59.99674691	0.810426155	ctx_lh_cuneus
-21.87644009	14.07402074	-47.78801843	ctx_lh_entorhinal
-30.9386489	-27.44044884	-34.66050092	ctx_lh_fusiform
-38.44759259	-46.19018519	10.055	ctx_lh_inferiorparietal
-45.16522307	-13.35528397	-36.35213969	ctx_lh_inferiortemporal
-6.797138047	-26.52777778	-0.606060606	ctx_lh_isthmuscingulate
-28.81817994	-68.91491475	-14.09027893	ctx_lh_lateraloccipital
-21.51043029	49.65374052	-35.04103453	ctx_lh_lateralorbitofrontal
-12.85870064	-50.95184803	-21.89164371	ctx_lh_lingual
-5.424302789	52.46314741	-33.96962151	ctx_lh_medialorbitofrontal
-51.38255075	-1.05607361	-29.19242037	ctx_lh_middletemporal
-20.70094021	-10.09763742	-33.50470106	ctx_lh_parahippocampal
-6.551643921	-8.080024814	28.71665633	ctx_lh_paracentral
-39.82481355	38.1677284	-3.969468614	ctx_lh_parsopercularis
-37.70623742	60.72183099	-31.45120724	ctx_lh_parsorbitalis
-41.22619629	47.33825684	-19.5916748	ctx_lh_parstriangularis
-11.2699553	-63.45146871	-10.27378672	ctx_lh_pericalcarine
-37.7076791	-0.500245339	17.57396958	ctx_lh_postcentral
-4.185311284	-0.283722438	12.20330739	ctx_lh_posteriorcingulate
-35.30378452	13.60621618	15.33101499	ctx_lh_precentral
-8.750484872	-38.07336113	13.38877036	ctx_lh_precuneus
-3.369775542	54.56656347	-17.64705882	ctx_lh_rostralanteriorcingulate
-27.43047381	65.73664656	-9.245512066	ctx_lh_rostralmiddlefrontal

X	Y	Z	Region
-27.43047381	65.73664656	-9.245512066	ctx_lh_rostralmiddlefrontal
-8.409821118	46.32435307	13.8764545	ctx_lh_superiorfrontal
-21.121497	-41.68930512	23.69906348	ctx_lh_superiorparietal
-46.72014687	8.292424968	-21.74289591	ctx_lh_superiortemporal
-46.00677227	-13.25964187	10.55130854	ctx_lh_supramarginal
-5.798690205	78.87528474	-29.70529613	ctx_lh_frontalpole
-25.94376504	31.79330393	-52.99268244	ctx_lh_temporalpole
-36.91558442	-0.342938312	-11.6599026	ctx_lh_transversetemporal
-30.75487945	20.2217279	-20.08969575	ctx_lh_insula
48.03367217	-19.09704574	-10.55749682	ctx_rh_bankssts
5.245158888	35.7336147	3.08031281	ctx_rh_caudalanteriorcingulate
31.32239106	30.37378154	20.14012328	ctx_rh_caudalmiddlefrontal
5.413062284	-61.50735294	2.649221453	ctx_rh_cuneus
19.14095007	13.38072122	-47.39684466	ctx_rh_entorhinal
30.54333274	-25.02878436	-34.88151553	ctx_rh_fusiform
40.10512232	-41.90762888	9.178134557	ctx_rh_inferiorparietal
43.5415707	-8.394461078	-39.20629894	ctx_rh_inferiortemporal
5.855446927	-27.09696728	-1.528830806	ctx_rh_isthmuscingulate
28.27539489	-65.8775836	-18.00317174	ctx_rh_lateraloccipital
21.03270609	48.73015873	-34.47388633	ctx_rh_lateralorbitofrontal
9.887762593	-49.87773888	-19.71100632	ctx_rh_lingual
4.966974573	55.82042114	-33.24071315	ctx_rh_medialorbitofrontal
50.85591502	-4.637917276	-30.79573414	ctx_rh_middletemporal
21.59495927	-9.769602851	-33.93711813	ctx_rh_parahippocampal
5.574623858	-6.455534659	29.1560317	ctx_rh_paracentral
43.43193444	33.05039788	-5.577159909	ctx_rh_parsopercularis
39.84943842	55.80170411	-30.06438807	ctx_rh_parsorbitalis
44.60538203	44.68959732	-16.17094734	ctx_rh_parstriangularis
10.07145966	-62.11767266	-9.486723738	ctx_rh_pericalcarine
37.2218269	-2.29390681	18.21908602	ctx_rh_postcentral
4.539522514	1.299480455	13.94575705	ctx_rh_posteriorcingulate
35.74047669	11.18046754	17.23375229	ctx_rh_precentral
7.952909887	-39.13619703	12.41339621	ctx_rh_precuneus
5.23004886	53.77137622	-18.7896987	ctx_rh_rostralanteriorcingulate
29.98936817	63.1596294	-6.599787363	ctx_rh_rostralmiddlefrontal
10.21130626	44.56652488	15.72779455	ctx_rh_superiorfrontal
19.52022891	-44.67677003	23.1629292	ctx_rh_superiorparietal
47.22200315	10.34015441	-23.27712519	ctx_rh_superiortemporal
46.5050845	-10.93363136	9.673192179	ctx_rh_supramarginal
7.123893805	80.40376106	-25.63053097	ctx_rh_frontalpole
22.85859073	30.3030888	-51.48117761	ctx_rh_temporalpole
38.81806931	1.170792079	-13.0730198	ctx_rh_transversetemporal
32.27828362	19.71071121	-21.13080728	ctx_rh_insula

S.10: Sample Average Entropy as Group Entropy

Theorem 1

Let H_{opt} be the group graph entropy for a unique state (task or no-task). Let H_k be the graph entropy for for k^{th} subject. We show that the sequence $\frac{1}{n}\sum_{k=1}^{n} H_k \rightarrow H_{opt}$ in probability. In other words, for any arbitrary small $\varepsilon > 0$

$$\lim_{n\to\infty} P(|H_{opt} - \frac{1}{n}\sum_{k=1}^{n} H_k| > \varepsilon) = 0 \tag{1}$$

Proof. Let Σ_{opt} be the corresponding adjacency matrix for H_{opt}. Here, the edge between regions (i, j) is given by $e_{i,j} = \sigma_{i,j} = \Sigma_{opt}(i, j)$. Let $G = (V, E)$, thus

$$q_{i,j} = \begin{cases} \frac{e_{ij}}{\Sigma(e_{ij}\in E)} \text{ when } i \neq j \\ 0 \text{ when } i = j \end{cases}$$

$$H_{opt} = \sum_{i,j} q_{i,j} \log_2(q_{i,j})$$

For k^{th} subject,

$$H_k = \sum_{i,j} q^k_{i,j} \log_2(q^k_{i,j})$$

It is clear that

$$\begin{aligned} |H_{opt} - \frac{1}{n}\sum_{k=1}^{n} H_k| &= |\sum_{i,j} q_{i,j}\log_2(q_{i,j}) - \frac{1}{n}\sum_{k=1}^{n}\sum_{i,j} q^k_{i,j}\log_2(q^k_{i,j})| \\ &= |\sum_{i,j}(q_{i,j}\log_2(q_{i,j}) - \frac{1}{n}\sum_{k=1}^{n} q^k_{i,j}\log_2(q^k_{i,j}))| \\ &\leq \sum_{i,j}|(q_{i,j}\log_2(q_{i,j}) - \frac{1}{n}\sum_{k=1}^{n} q^k_{i,j}\log_2(q^k_{i,j}))| \text{ according to cauchy schwarz inequality} \end{aligned} \tag{2}$$

From, Eqn. 2 and 1,

$$\lim_{n\to\infty} P(\sum_{i,j}|(q_{i,j}\log_2(q_{i,j}) - \frac{1}{n}\sum_{k=1}^{n} q^k_{i,j}\log_2(q^k_{i,j}))| > \varepsilon) = 0 \rightarrow \lim_{n\to\infty} P(|H_{opt} - \frac{1}{n}\sum_{k=1}^{n} H_k| > \varepsilon) = 0 \tag{3}$$

Let's see the result for (i, j). Using log-sum inequality,

$$\begin{aligned} &\frac{1}{n}\sum_{k=1}^{n} q^k_{i,j}\log_2(q^k_{i,j}) \geq \frac{1}{n}\sum_{k=1}^{n} q^k_{i,j}\log_2\frac{(\sum_{k=1}^{n} q^k_{i,j})}{n} \\ &\text{or, } -\frac{1}{n}\sum_{k=1}^{n} q^k_{i,j}\log_2(q^k_{i,j}) \leq -\frac{1}{n}\sum_{k=1}^{n} q^k_{i,j}\log_2\frac{(\sum_{k=1}^{n} q^k_{i,j})}{n} \\ &\text{or, } |q_{i,j}\log_2(q_{i,j}) - \frac{1}{n}\sum_{k=1}^{n} q^k_{i,j}\log_2(q^k_{i,j})| < |q_{i,j}\log_2(q_{i,j}) - \frac{1}{n}\sum_{k=1}^{n} q^k_{i,j}\log_2\frac{(\sum_{k=1}^{n} q^k_{i,j})}{n})| \end{aligned} \tag{4}$$

For simplicity let's define $z = q_{i,j}$ and $z_n = \frac{1}{n}\sum_{k=1}^{n} q^k_{i,j}$. Also, let $G : (0, 1] \rightarrow R$ such that $G(z) = z\log_2(z)$. Clearly G is a continuous function in the range of z.

Using law of law of large numbers $z_n \rightarrow z$ in probability. Now continuous mapping theorem implies $G(z_n) \rightarrow G(z)$ in probability.

Let $z^k = q^k_{i,j}$, **we need to prove that** $\frac{1}{n}\sum_{k=1}^{n} G(z_k) \rightarrow G(z)$ in probability.
As $G(z_n) \rightarrow G(z)$ in probability,

$$\lim_{n\to\infty} P(|G(z) - G(z_n)| > \varepsilon) = 0 \tag{5}$$

From Eqn. 4,

$$|G(z) - G(z_n)| > |G(z) - \frac{1}{n}\sum_{k=1}^{n} G(z_k)|$$

Eqn. 5 implies as $n \rightarrow \infty$,

$$\begin{aligned}&|G(z) - G(z_n)| < \varepsilon \\ &\text{or, } |G(z) - \frac{1}{n}\sum_{k=1}^{n} G(z_k)| < |G(z) - G(z_n)| < \varepsilon\end{aligned}$$

Hence,

$$\lim_{n\to\infty} P(|G(z) - \frac{1}{n}\sum_{k=1}^{n} G(z_k)| > \varepsilon) = 0 \tag{6}$$

Hence we prove that,

$$\lim_{n\to\infty} P(\sum_{i,j} |(q_{i,j}\log_2(q_{i,j}) - \frac{1}{n}\sum_{k=1}^{n} q^k_{i,j}\log_2(q^k_{i,j}))| > K\varepsilon) = 0 \text{ where K is number of edges (constant)}$$

Using the same inequality, it follows that,

$$\lim_{n\to\infty} P(|H_{opt} - \frac{1}{n}\sum_{k=1}^{n} H_k| > \varepsilon) = 0$$

where $K\varepsilon$ is replaced as ε. □

As a corollary, mean value of subject *sub-graph* entropies (H^r_k) also approaches group *sub-graph* (H^r_{opt}) entropy in probability, *i.e.*, for region r.

$$\lim_{n\to\infty} P(|H^r_{opt} - \frac{1}{n}\sum_{k=1}^{n} H^r_k| > \varepsilon) = 0$$

S.11: Maximizing *Sub-Graph* Entropy is Equivalent to Maximizing Mutual Information

Proof. Notice that once we know the communication pattern for the whole graph, we also know the communication pattern for the *sub-graph*s. So, given G, the conditional entropy of *sub-graph* G_s given by $H(G_s|G)$ equals 0.

$$\begin{aligned}I(G;G_{v_s}) &= H(G_s) - H(G_s|G) \\ &= H(G_s), \text{ as } H(G_s|G) = 0\end{aligned}$$

□

S.12: Correlation Coefficient as Edge Weight

Let x denote the fMRI time series from R regions of a subject for a state (task or no-task). It has size $R \times 1$ where R is regions. Also, let x_r be the time series from r^{th} region.

Theorem 2
Let the edge weight $e_{i,j} \sim Ber(\theta_{i,j})$ where $Ber(\theta)$ is Bernoulli distribution with parameter θ. Here $\theta_{i,j}$ captures the relationship between regions (i,j). Assume that, $\mathbf{x} \sim N(0,\Sigma)$ where Σ is covariance matrix with $\Sigma(i,j) = \sigma_{i,j}$, i.e., $\sigma_{i,j}$ is $(i,j)^{th}$ entry of Σ. Then the minimum mean square error (MMSE) estimate of $e_{i,j}$ is given by $\hat{e}_{i,j} = \sigma_{i,j}$.

Proof. The $(i,j)^{th}$ entry of Σ is given by $\sigma_{i,j} = E[x_i x_j]$. As x is Gaussian, the covariance captures all the information between the relation of two regions (i,j). Hence, $\theta_{i,j} = \sigma_{i,j}$.

To calculate the MMSE estimate, we note that $P(e_{i,j}) = \begin{cases} \theta_{i,j} \; if \; e_{i,j} = 1 \\ 1-\theta_{i,j} \; if \; e_{i,j} = 0 \end{cases}$

In order to calculate the estimation, we minimize $E[(\hat{e}_{i,j} - e_{i,j})^2]$.

$$\begin{aligned} E[(\hat{e}_{i,j} - e_{i,j})^2] &= (1-\hat{e}_{i,j})^2\theta + (0-\hat{e}_{i,j})^2(1-\theta) = (1-2\hat{e}_{i,j})\theta + \hat{e}_{i,j}^2 \\ &= (\hat{e}_{i,j} - \theta)^2 + \theta(1-\theta) \geq (\hat{e}_{i,j} - \theta)^2 \text{ as } \theta \in [0,1]. \end{aligned}$$

Also, $E[(\hat{e}_{i,j} - e_{i,j})^2] \geq 0$. So, the value for which MMSE is minimum is achievable by $\hat{e}_{i,j}$ equal to $\theta_{i,j}$. Thus, $\hat{e}_{i,j} = \theta_{i,j} = \sigma_{i,j}$. □
